# Lineage-specific silencing of *PSAT1* induces serine auxotrophy and sensitivity to dietary serine starvation in luminal breast tumors

**DOI:** 10.1016/j.celrep.2021.110278

**Published:** 2022-01-18

**Authors:** Bo-Hyun Choi, Vipin Rawat, Jenny Högström, Philippa A. Burns, Kelly O. Conger, Mete Emir Ozgurses, Jaymin M. Patel, Tejas S. Mehta, Angelica Warren, Laura M. Selfors, Taru Muranen, Jonathan L. Coloff

**Affiliations:** 1 Department of Physiology and Biophysics, University of Illinois College of Medicine, 835 South Wolcott Avenue, Room E202 M/C 901, Chicago, IL 60612, USA; 2 University of Illinois Cancer Center, Chicago, IL 60612, USA; 3 Beth Israel Deaconess Medical Center, Boston, MA 02215, USA; 4 Department of Cell Biology, Harvard Medical School, Boston, MA 02115, USA; 5 Department of Radiology, UMass Memorial Medical Center, Worcester, MA 01655, USA; 6 Present address: Department of Pharmacology, School of Medicine, Daegu Catholic University, Daegu 42472, Republic of Korea; 7 Lead contact

## Abstract

A major challenge of targeting metabolism for cancer therapy is pathway redundancy, in which multiple sources of critical nutrients can limit the effectiveness of some metabolism-targeted therapies. Here, we analyze lineage-dependent gene expression in human breast tumors to identify differences in metabolic gene expression that may limit pathway redundancy and create therapeutic vulnerabilities. We find that the serine synthesis pathway gene *PSAT1* is the most depleted metabolic gene in luminal breast tumors relative to basal tumors. Low PSAT1 prevents *de novo* serine biosynthesis and sensitizes luminal breast cancer cells to serine and glycine starvation *in vitro* and *in vivo*. This *PSAT1* expression disparity preexists in the putative cells of origin of basal and luminal tumors and is due to luminal-specific hypermethylation of the *PSAT1* gene. Our data demonstrate that luminal breast tumors are auxotrophic for serine and may be uniquely sensitive to therapies targeting serine availability.

## In brief

Many cancer types can synthesize serine *de novo*, which limits the effectiveness of dietary serine starvation. Choi et al. demonstrate that luminal breast tumors are auxotrophic for serine due to lineage-specific hypermethylation of the *PSAT1* gene. This serine auxotrophy may be a targetable vulnerability of luminal breast tumors.

## Graphical abstract

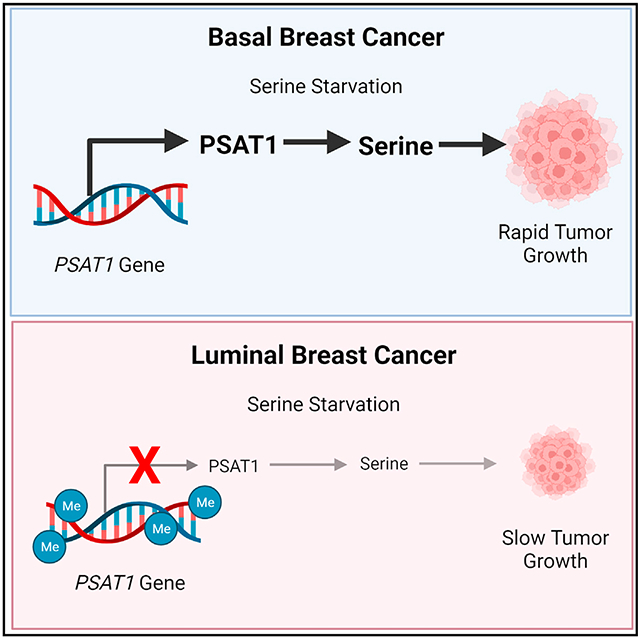

## INTRODUCTION

The unique metabolic phenotypes observed in cancer cells are driven by numerous factors, including genetic driver mutations, nutrient and oxygen availability, and the proliferation rate of the tumor (Vander Heiden and DeBerardinis, 2017). Recent work has also highlighted the extent to which tumor lineage (i.e., the cell or tissue of origin) contributes to metabolic phenotypes in cancer (Mayers and Vander Heiden, 2017). This has been observed in human tumors at the gene expression level (Gaude and Frezza, 2016; Hu et al., 2013) and in controlled experiments demonstrating that the same driver mutations can induce distinct metabolic phenotypes depending on the tissue type in which they are activated (Mayers et al., 2016; Yuneva et al., 2012). The impact of tumor lineage on cancer phenotypes has also been emphasized by recent projects summarizing The Cancer Genome Atlas (TCGA) project, in which a primary conclusion is that cell-of-origin gene expression patterns dominate the molecular classification of most tumors (Hoadley et al., 2018; Selfors et al., 2017).

Breast cancer is a highly heterogeneous disease made up of distinct subtypes of tumors. In the clinic, the histological expression of the estrogen receptor (ER), progesterone receptor (PR), and the receptor tyrosine kinase human epidermal growth factor receptor 2 (HER2) have guided therapeutic decisions for decades. Gene expression profiles have also been used to identify at least five molecular subtypes of breast cancer–basal, luminal A, luminal B, HER2+, and normal-like (Curtis et al., 2012; Lehmann et al., 2011; Perou et al., 2000; Sorlie et al., 2001). These molecular subtypes largely overlap with the clinical subtypes, with most luminal tumors being positive for ER and/or PR, and most basal tumors being triple negative (i.e., negative for ER, PR, and HER2) (The Cancer Genome Atlas Network, 2012). More recently, molecular landscaping projects have simplified the classification of breast tumors into two primary lineages–luminal and basal–that are as distinct from each other as they are from tumors arising from completely distinct tissues of origin (Hoadley et al., 2018). When detected early, luminal tumors have a more favorable prognosis than basal tumors, in part due to their responsiveness to endocrine therapies; however, many luminal breast cancer patients suffer from relapse due to the development of therapeutic resistance (Osborne and Schiff, 2011). While the recent approvals of CDK4/6 and phosphatidylinositol 3-kinase (PI3K) inhibitors have improved the treatment of advanced luminal breast cancer, these therapies are still not curative for most patients (Andre et al., 2019; Finn et al., 2016; Goetz et al., 2017; Hortobagyi et al., 2018). As a result, luminal breast tumors continue to account for approximately half of all breast cancer fatalities (Siegel et al., 2019).

While recent research has identified many potential metabolic targets for cancer therapy, pathway redundancy has emerged as a major challenge of targeting metabolism. The human genome, and in particular the human metabolic network, contains a high degree of functional redundancy (Nowak et al., 1997; Thiele et al., 2013). This plasticity is beneficial at the organismal level by allowing adaptation in a changing nutrient environment, but it complicates attempts to target metabolic pathways for cancer therapy. Here, we took the approach of analyzing metabolic gene expression in human breast tumors to identify cases in which redundancy may be limited by lineage-dependent gene expression, thereby creating a metabolic vulnerability. Using this approach, we have found that the most significant differences in metabolic gene expression between luminal and basal breast tumors are found in the serine synthesis pathway, especially phosphoserine aminotransferase 1 (*PSAT1*), which is expressed at far lower levels in luminal tumors than basal tumors. Serine is a non-essential amino acid that is important for cancer cell proliferation not only for protein synthesis but also for the synthesis of other amino acids, nucleotides, lipids, and antioxidants (Mattaini et al., 2016). Serine can be either taken up from the circulation or synthesized *de novo* via a three-step process catalyzed by phosphoglycerate dehydrogenase (PHGDH), PSAT1, and phosphoserine phosphatase (PSPH). Importantly, *PHGDH* amplifications and an enhanced ability to synthesize serine have previously been reported in basal breast tumors (Locasale et al., 2011; Possemato et al., 2011). This and other work motivated the development of PHGDH inhibitors as potential cancer treatments (Mullarky et al., 2016; Pacold et al., 2016; Rohde et al., 2018; Wang et al., 2017). While the inhibition of serine synthesis has shown efficacy in some models and is still being evaluated, it is becoming clear that in some circumstances it is not effective because of extracellular serine that can be taken up to offset the inhibition of *de novo* biosynthesis (Chen et al., 2013; Montrose et al., 2021; Méndez-Lucas et al., 2020; Ngo et al., 2020; Nilsson et al., 2012; Sullivan et al., 2019b; Tajan et al., 2021). Because our goal was to identify cases in which lineage-dependent gene expression reduces pathway redundancy, we examined whether the very low expression of *PSAT1* found in luminal tumors limits their ability to synthesize serine and creates a dependence on exogenous serine for growth. We have found that luminal breast cancer cells are auxotrophic for serine because of lineage-specific hypermethylation of the *PSAT1* gene and are sensitive to serine starvation both *in vitro* and *in vivo*.

## RESULTS

### Lineage-specific suppression of the serine synthesis pathway in luminal breast cancer

**Figure 1.**
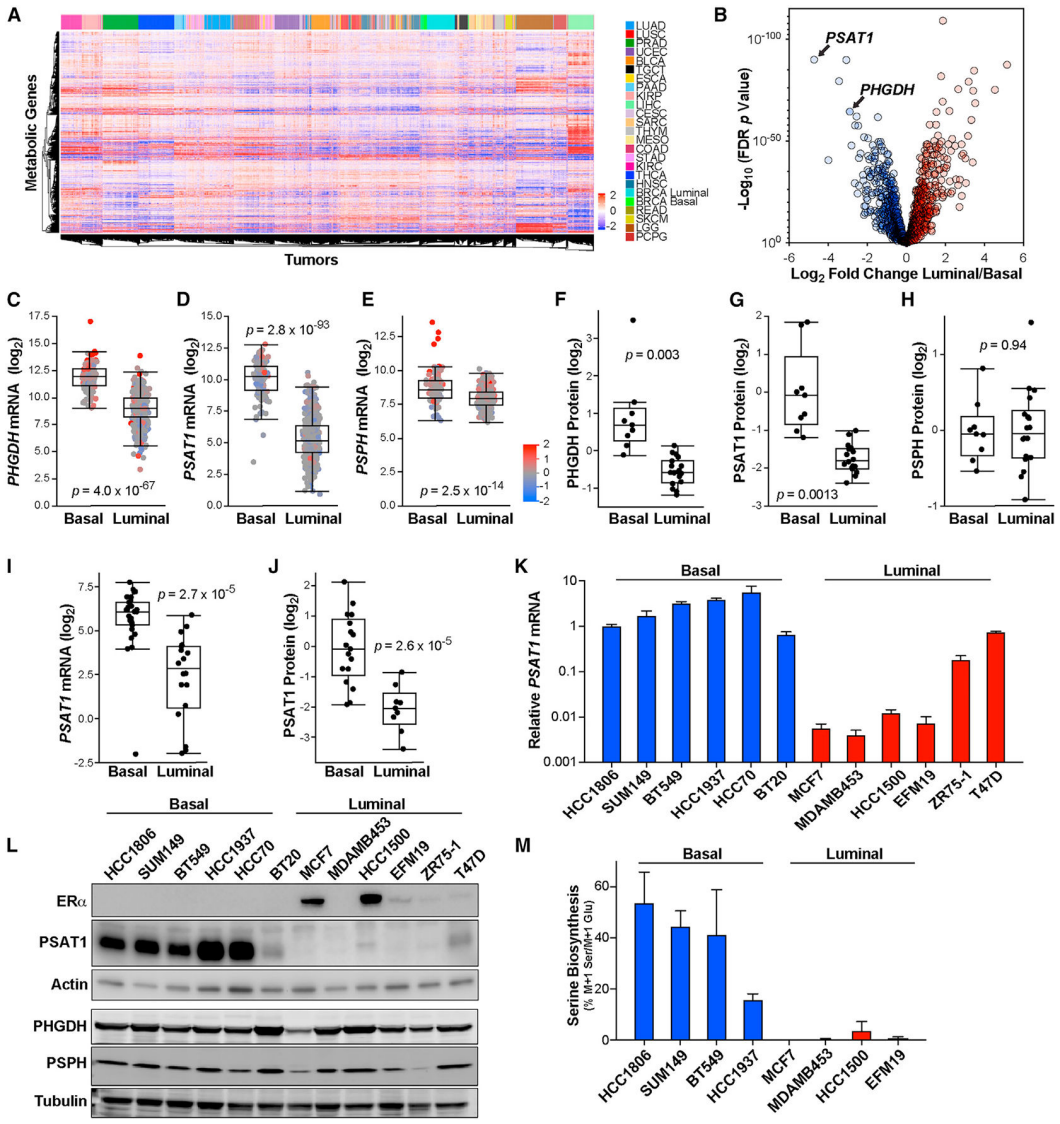
Lineage-specific suppression of the serine synthesis pathway in luminal breast tumors (A) Hierarchical clustering of human tumors from the TCGA Pan-Cancer Atlas dataset by metabolic gene expression. Data are log_2_ median centered. TCGA study abbreviations: LUAD, lung adenocarcinoma; LUSC, lung squamous cell carcinoma; PRAD, prostate adenocarcinoma; UCEC, uterine corpus endometrial carcinoma; BLCA, bladder urothelial carcinoma; TGCT, testicular germ cell tumors; ESCA, esophageal carcinoma; PAAD, pancreatic adenocarcinoma; KIRP, kidney renal papillary cell carcinoma; LIHC, liver hepatocellular carcinoma; CESC, cervical squamous cell carcinoma and endocervical adenocarcinoma; SARC, sarcoma; THYM, thymoma; MESO, mesothelioma; COAD, colon adenocarcinoma; STAD, stomach adenocarcinoma; KIRC, kidney renal clear cell carcinoma; THCA, thyroid carcinoma; HNSC, head and neck squamous cell carcinoma; BRCA, breast invasive carcinoma; READ, rectum adenocarcinoma; SKCM, skin cutaneous melanoma; LGG, brain lower grade glioma; PCPG, pheochromocytoma and paraganglioma. (B) Differences in metabolic gene expression between luminal and basal breast tumors in the TCGA Pan-Cancer Atlas RNA sequencing (RNA-seq) dataset. Data are the log_2_ fold change of mean gene expression in luminal breast tumors relative to basal breast tumors. −Log_10_ p values from 2-sided Welch’s t tests that have been corrected for false discovery using the Benjamani-Hochberg method. (C–E) mRNA levels of *PHGDH* (C), *PSAT1* (D), and *PSPH* (E) in basal and luminal breast tumors in TCGA data. p values from 2-sided Welch’s t tests. Tumors are colored based on gene copy-number data. (F–H) Protein levels of PHGDH (F), PSAT1 (G), and PSPH (H) in basal and luminal breast tumors in proteome analyses. p values from 2-sided Welch’s t tests. (I and J) mRNA (I) and protein (J) levels of PSAT1 in basal and luminal breast cancer cell lines from the Cancer Cell Line Encyclopedia (CCLE). p values from 2-sided Welch’s t tests. (K) *PSAT1* mRNA level in basal and luminal breast cancer cell lines. Values are the means ± SEMs of 3 independent experiments. (L) Representative western blot of ERα, PHGDH, PSAT1, and PSPH in basal and luminal breast cancer cell lines. (M) Serine biosynthesis in basal and luminal breast cancer cell lines. Values are the means ± SEMs of 2 independent experiments.

To evaluate the relationship between tumor lineage and metabolic gene expression, we analyzed the expression of a curated list of 1,454 metabolic genes (Gaude and Frezza, 2016) in the TCGA Pan-Cancer Atlas dataset (Hoadley et al., 2018). Unsupervised hierarchical clustering of metabolic genes in the 24 largest tumor types revealed that metabolic gene expression alone was largely sufficient to distinguish tumor tissue of origin (Figure 1A). Analyzing metabolic gene expression in breast tumors alone showed a clear distinction of the basal subtype, while luminal A and luminal B tumors largely clustered together (Figure S1A). Because luminal A and B tumors were similar with respect to metabolic gene expression, we focused our further analyses on luminal (i.e., luminal A + luminal B) and basal breast tumors, which cluster distinctly in our pan-cancer analysis of metabolic gene expression (Figure 1A).

To focus on potential vulnerabilities created by lineage-dependent gene expression, we identified the most significant gene expression differences between luminal and basal tumors (Figure 1B). This analysis found that *PSAT1* is the most depleted metabolic gene, with luminal tumors expressing 26-fold less *PSAT1* than basal tumors (Figures 1B and 1D). *PHGDH* was also among the top-scoring genes, and *PSPH* expression was also reduced in luminal breast tumors (Figures 1B, 1C, and 1E). Similar results were found in another large human breast tumor dataset (Figure S1B) (Curtis et al., 2012). Notably, these differences are not solely due to copy-number alterations, as most tumors display these phenotypes in the absence of amplifications or deletions (Figures 1C-1E). Analysis of the proteome of human breast tumors (Johansson et al., 2019) also revealed very low levels of PHGDH and PSAT1 (but not PSPH) protein in luminal breast tumors (Figures 1F-1H). Using data from the Cancer Cell Line Encyclopedia (CCLE) (Barretina et al., 2012) and our own qPCR and western blots, we found that breast cancer cell lines robustly maintain the low-*PSAT1* phenotype of luminal tumors, while the differences in *PHGDH* and *PSPH* were less pronounced or absent (Figures 1I-1L and S1C-S1H). To determine whether these gene expression differences result in relevant metabolic changes, we determined the percentage of intracellular serine that is synthesized in the serine synthesis pathway by tracing the incorporation of ^15^N from α-^15^N-glutamine into serine. Because nitrogen from glutamate is used by PSAT1 to generate serine, normalization of M + 1 serine to M + 1 glutamate describes an accurate fraction of intracellular serine made in the serine synthesis pathway. Consistent with our expression analyses, we detected high serine synthesis pathway activity in basal, but not luminal, cell lines (Figure 1M).

### Luminal breast cancer cells are auxotrophic for serine

We next sought to determine whether luminal and basal breast cancer cells differ in their response to serine starvation, which inhibits the growth of some cancer cells *in vitro* and *in vivo* (Maddocks et al., 2013, 2017; Muthusamy et al., 2020). Glycine is typically removed in these experiments because some tissues can convert glycine back to serine *in vivo*, even though glycine cannot replace serine to support cancer cell proliferation (Labuschagne et al., 2014). Because the non-physiological nutrient levels found in traditional tissue culture medium can affect cellular dependence on certain amino acids (Muir et al., 2017; Tardito et al., 2015), we first determined whether physiological medium (human plasma-like medium [HPLM]) (Cantor et al., 2017) affects the response to serine and glycine (S/G) starvation. While breast cancer cells proliferate slightly faster in RPMI than HPLM in the presence of S/G, upon S/G starvation, cells proliferate much more slowly in RPMI than HPLM (Figures S2A-S2D), indicating that the use of physiological medium allows for enhanced growth without S/G, as has recently been noted (Hennequart et al., 2021). While at present we do not fully understand the cause of these differences, we used the more physiologically relevant HPLM for the remainder of our experiments, with the exception of the organoid work described in Figure 4.

**Figure 2.**
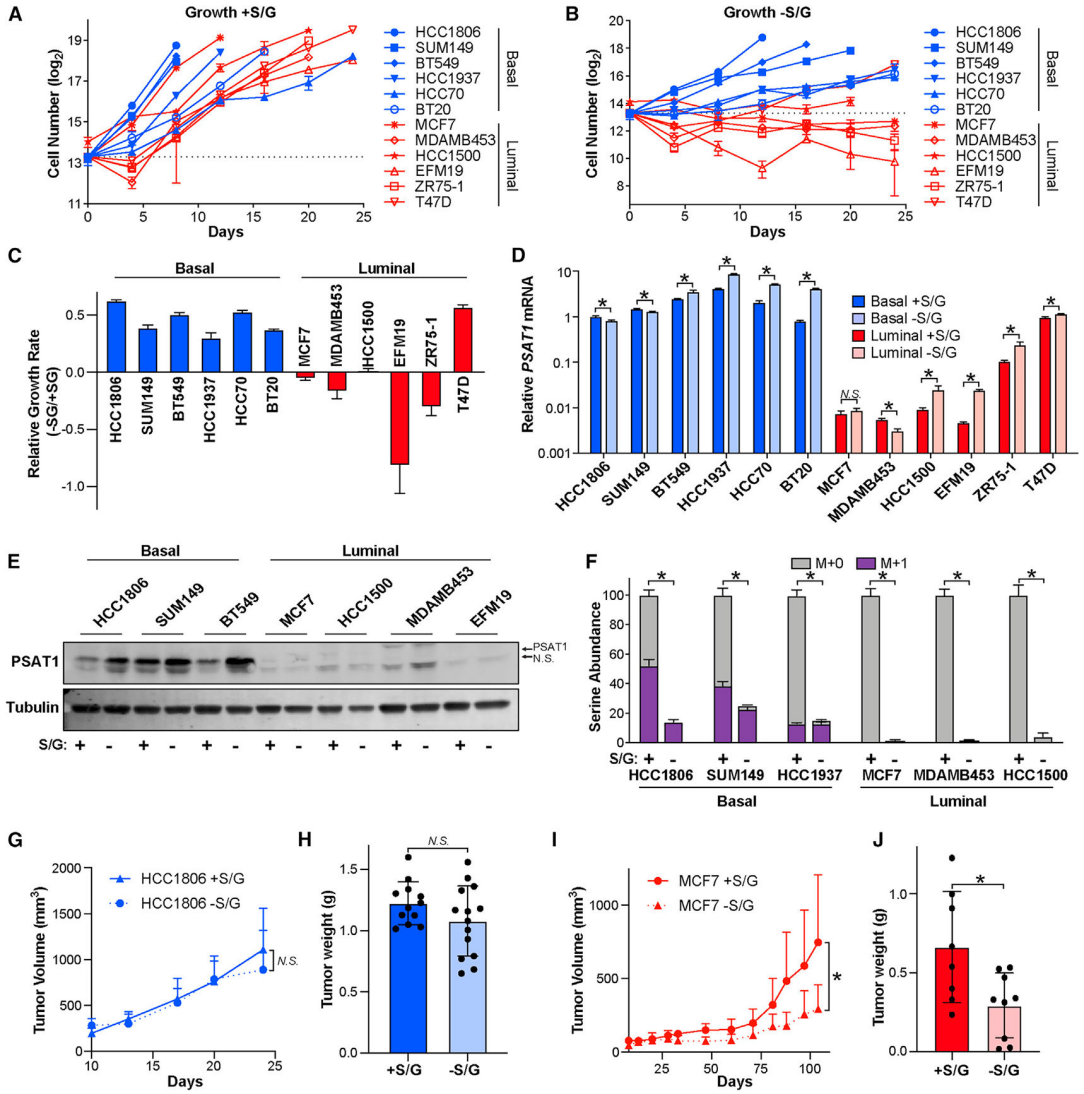
Luminal breast cancer cells are auxotrophic for serine (A and B) Basal and luminal breast cancer cell lines were cultured in normal HPLM media (+S/G) (A) or HPLM without S/G (−S/G) (B), and cell numbers were counted for 24 days or until cells became confluent. S/G starvation began on day 2. The dashed line indicates the number of cells plated per well. (C) Relative growth rates of basal and luminal breast cancer cells grown in the presence and absence of S/G. (D) *PSAT1* mRNA level in basal and luminal lines treated with and without S/G for 48 h. Values are the means ± SDs of triplicate samples from an experiment representative of 3 independent experiments. *p < 0.05 from unpaired 2-sided t tests. N.S. (not significant) indicates p > 0.05. (E) Representative western blot of basal and luminal cell lines treated with and without S/G for 48 h. (F) Serine abundance and biosynthesis in basal and luminal lines treated with and without S/G for 48 h. M + 1 (in purple) indicates “heavy” serine made in the serine synthesis pathway. Values are the means ± SDs of triplicate samples from an experiment representative of 2 independent experiments. *p < 0.05 from unpaired 2-sided t tests on serine abundance data. (G) Tumor volume over time after injecting HCC1806 cells into the mammary glands of nude mice fed with or without S/G diet. Values are the means ± SDs. n = 12 tumors for with S/G and n = 14 tumors for without S/G. N.S. indicates p > 0.05 in mixed-effects model analysis. (H) HCC1806 tumor weight as measured at endpoint. Values are the means ± SDs (n = 12 tumors for with S/G and n = 14 tumors for without S/G). N.S. indicates p > 0.05 in 2-sided Welch’s t test. (I) Tumor volume over time after injecting MCF7 cells into the mammary glands of nude mice fed with or without S/G diet. Values are the means ± SDs (n = 8 tumors for with S/G and n = 9 tumors for without S/G). *p < 0.05 in mixed-effects model analysis. (J) MCF7 tumor weight as measured at the endpoint. Values are the means ± SDs (n = 8 tumors for with S/G and n = 9 tumors for without S/G). *p < 0.05 in 2-sided Welch’s t test.

We performed S/G starvation on 6 basal (HCC1806, SUM149, BT549, HCC1937, HCC70, and BT20) and 6 luminal cancer cell lines (MCF7, MDAMB453, ZR75-1, EFM19, HCC1500, and T47D), in which we found that all cell lines grow more slowly in the absence of S/G (Figures 2A-2C, S2E, and S2F). Importantly, however, all basal cell lines were able to proliferate without S/G, while only one luminal cell line (T47D) showed this capability (Figures 2B, 2C, S2E, and S2F). Interestingly, T47D cells have relatively high *PSAT1* expression that is more comparable to basal cell lines than other luminal lines (Figures 1K and 1L). S/G starvation increased serine synthesis pathway gene expression in most cell lines, but PSAT1 mRNA and protein remained very low in most luminal lines (Figures 2D, 2E, S2G, and S2H). Moreover, long-term (30-day) S/G deprivation did not further increase *PSAT1* expression, indicating that the low *PSAT1* phenotype of luminal cells is durable under long-term selective pressure (Figure S2I). In agreement with published results (Labuschagne et al., 2014), we found that serine starvation alone caused the vast majority of the growth inhibitory effects on luminal cells, with glycine being insufficient to support proliferation (Figures S2J and S2K). We also found that luminal breast cancer cells demonstrate a dramatic reduction in intracellular serine upon S/G starvation, while basal cells are able to maintain a much larger serine pool (Figure 2F). Consistently, ^15^N tracing revealed that most of the serine found in basal cells cultured without S/G in the medium was made in the serine synthesis pathway and that *de novo* serine biosynthesis remained virtually undetectable in luminal cells grown in the absence of S/G (Figure 2F). These results indicate that most luminal breast cancer cell lines are auxotrophic for serine and are highly dependent on exogenous serine to support proliferation.

To determine whether the difference in basal and luminal cancer cell dependence on exogenous serine is recapitulated in the more complex *in vivo* environment, we injected basal HCC1806 and luminal MCF7 cells orthotopically into the mammary fat pads of nude mice and fed them custom diets with and without S/G. Consistent with previous reports, we found that an S/G-free diet reduces plasma serine and glycine levels by ~50% (Figures S2L and S2M). In line with our *in vitro* findings, the S/G-free diet had no effect on basal HCC1806 tumor growth but significantly inhibited the growth of luminal MCF7 tumors (Figures 2G-2J).

### PSAT1 expression determines growth in the absence of exogenous serine

**Figure 3.**
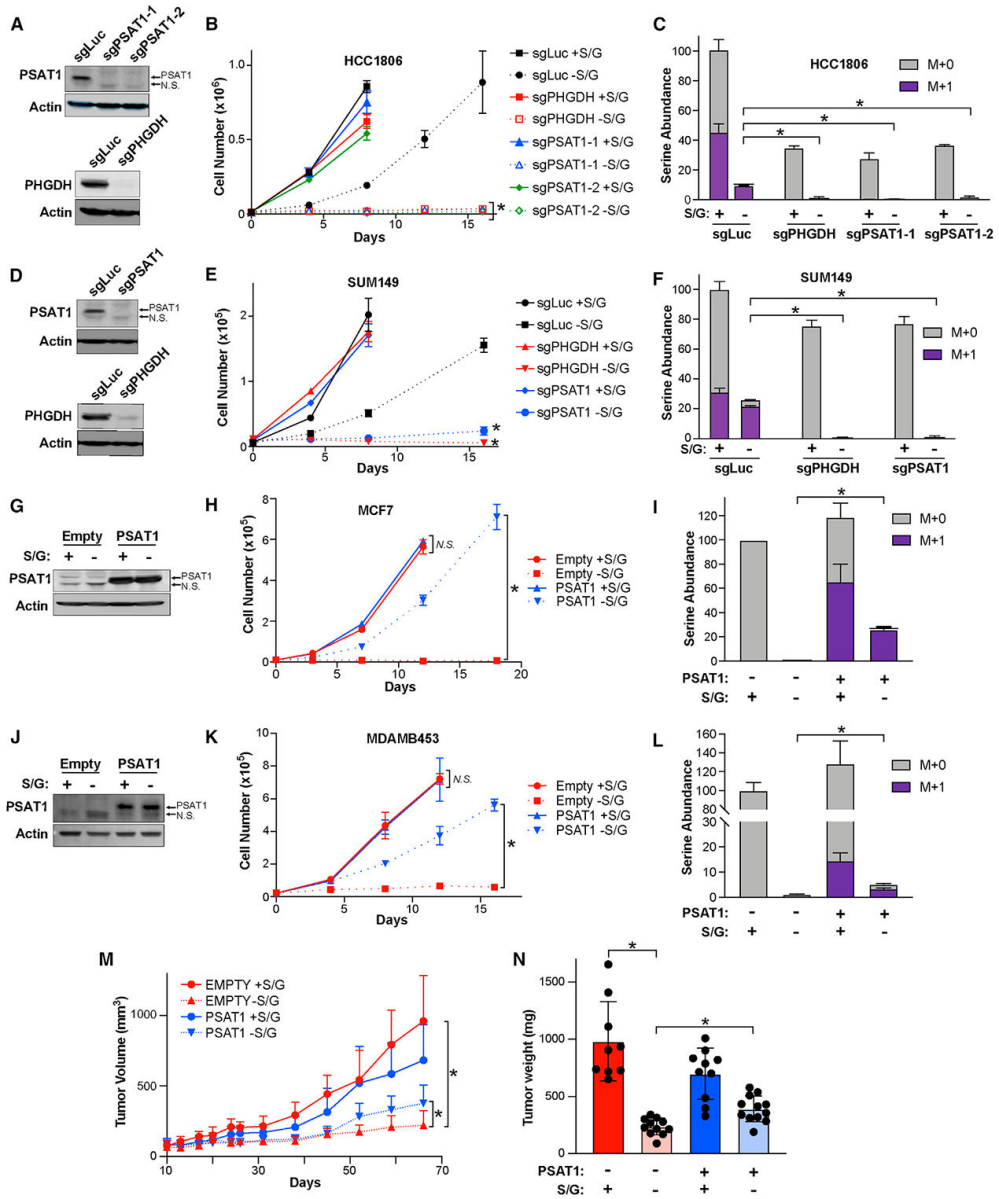
PSAT1 expression determines growth in the absence of exogenous serine (A) Representative western blots of control (sgLuc), PSAT1 knockout (sgPSAT1-1 and sgPSAT1-2), and PHGDH knockout (sgPHGDH) HCC1806 cells. (B) Growth curve of control (sgLuc), sgPHGDH, and sgPSAT1 HCC1806 cells grown with or without S/G. Values are the means ± SDs of triplicate samples from an experiment representative of 3 independent experiments. *p < 0.05 in 2-way repeated measures ANOVA. sgPSAT1 or sgPHGDH without S/G data were compared to sgLuc without S/G data. (C) Serine abundance and biosynthesis in sgLuc, sgPHGDH, and sgPSAT1 HCC1806 cells treated with or without S/G for 48 h. M + 1 (in purple) indicates “heavy” serine made in the serine synthesis pathway. Values are the means ± SDs of triplicate samples from an experiment representative of 2 independent experiments. *p < 0.05 from unpaired 2-sided t tests on serine abundance data. (D) Representative western blots of control (sgLuc), PSAT1 knockout (sgPSAT1), and PHGDH knockout (sgPHGDH) SUM149 cells. (E) Growth curve of control (sgLuc), sgPHGDH, and sgPSAT1 SUM149 cells grown with or without S/G. Values are the means ± SDs of triplicate samples from an experiment representative of 3 independent experiments. *p < 0.05 in 2-way repeated measures ANOVA. sgPSAT1 or sgPHGDH without S/G data were compared to sgLuc without S/G data. (F) Serine abundance and biosynthesis in sgLuc, sgPHGDH, and sgPSAT1 SUM149 cells treated with or without S/G for 48 h. M + 1 (in purple) indicates “heavy” serine made in the serine synthesis pathway. Values are the means ± SDs of triplicate samples from an experiment representative of 2 independent experiments. *p < 0.05 from unpaired 2-sided t tests on serine abundance data. (G) Representative western blot of control (Empty) and PSAT1 overexpressing MCF7 cells (PSAT1) cultured with or without S/G for 48 h. (H) Growth curve of Empty and PSAT1 MCF7 cells cultured with or without S/G. Values are the means ± SDs of triplicate samples from an experiment representative of 3 independent experiments. *p < 0.05 and N.S. indicates p > 0.05 in 2-way repeated measures ANOVA tests comparing Empty and PSAT1 cells either with or without S/G. (I) Serine abundance and biosynthesis in Empty and PSAT1 MCF7 cells cultured with or without S/G for 48 h. M + 1 (in purple) indicates “heavy” serine made in the serine synthesis pathway. Values are the means ± SDs of triplicate samples from an experiment representative of 2 independent experiments. *p < 0.05 from unpaired 2-sided t tests on serine abundance data. (J) Representative western blot of control (Empty) and PSAT1 overexpressing MDAMB453 cells (PSAT1) with or without S/G for 48 h. (K) Growth curve of Empty and PSAT1 MDAMB453 cells cultured with or without S/G. Values are the means ± SDs of triplicate samples from an experiment representative of 3 independent experiments. *p < 0.05 and N.S. indicates p > 0.05 in 2-way repeated-measures ANOVA tests comparing Empty and PSAT1 cells either with or without S/G. (L) Serine abundance and biosynthesis in Empty and PSAT1 MDAMB453 cells cultured with or without S/G for 48 h. M + 1 (in purple) indicates “heavy” serine made in the serine synthesis pathway. Values are the means ± SDs of triplicate samples from an experiment representative of 2 independent experiments. *p < 0.05 from unpaired 2-sided t tests on serine abundance data. (M) Tumor volume over time after injecting Empty and PSAT1 MCF7 cells into the mammary gland of the nude mice fed with or without S/G diet. Values are the means ± SDs (n = 9 tumors for Empty with S/G, n = 11 tumors for Empty without S/G, n = 10 tumors for PSAT1 with S/G and n = 12 tumors for PSAT1 without S/G). *p < 0.05 in mixed-effects model analyses. (N) Empty and PSAT1 MCF7 xenograft tumor weight as measured at endpoint. Values are the means ± SDs (n = 9 tumors for Empty with S/G, n = 11 tumors for Empty without S/G, n = 10 tumors for PSAT1 with S/G and n = 12 tumors for PSAT1 without S/G). *p < 0.05 in 2-sided Welch’s t tests.

Previous studies have demonstrated that several genetic features of tumors influence the response to serine starvation (DeNicola et al., 2015; Kottakis et al., 2016; LeBoeuf et al., 2020; Maddocks et al., 2013, 2017). In addition, the ability to synthesize serine *de novo* can allow cancer cells to proliferate when exogenous serine is limiting (Diehl et al., 2019; Montrose et al., 2021; Méndez-Lucas et al., 2020; Ngo et al., 2020; Sullivan et al., 2019b; Tajan et al., 2021). We examined whether the ability of basal breast cancer cells to proliferate without exogenous S/G is reliant on serine biosynthesis by treating basal HCC1806 cells with the PHGDH inhibitor PH-755 while growing with and without S/G. Despite strongly inhibiting serine synthesis, PH-755 had no effect on growth when S/G are present, but it nearly completely blocked proliferation when extracellular S/G were absent (Figures S3A and S3B). Similarly, CRISPR-mediated knockout of either PHGDH or PSAT1 in HCC1806 and SUM149 cells had virtually no effect on proliferation in the presence of S/G but completely halted growth upon S/G starvation (Figures 3A, 3B, 3D, and 3E). Accordingly, knockout of PHGDH or PSAT1 prevented serine synthesis and the maintenance of a serine pool in basal cells cultured without S/G (Figures 3C and 3F).

To determine whether the low PSAT1 expression of luminal cells contributes to their inability to grow in the absence of S/G, we overexpressed PSAT1 in the luminal cell lines MCF7 and MDAMB453 (Figures 3G and 3J). While not affecting growth with S/G, PSAT1 overexpression was sufficient to allow luminal cell proliferation without S/G (Figures 3H and 3K). Furthermore, we found that PSAT1 overexpression was sufficient to allow *de novo* serine synthesis even in the presence of exogenous S/G and allowed for the maintenance of a relatively large serine pool upon S/G deprivation (Figures 3I and 3L). We also found that the inhibition of PHGDH prevented PSAT1-induced growth without S/G (Figure S3C) and that a catalytically inactive PSAT1 mutant (PSAT1 K200A) was not able to support growth without S/G (Figures S3D-S3F), indicating that this rescue was specifically due to the ability of PSAT1 to promote serine synthesis and not another unknown function. Furthermore, we found that the overexpression of PHGDH in MCF7 cells was not sufficient to promote serine biosynthesis or growth without S/G (Figures S3E-S3G), and that knockout of PSAT1 in T47D cells prevented their ability to grow without S/G (Figures S3H-S3J), demonstrating that PSAT1 is responsible for luminal cell growth upon serine starvation. Importantly, PSAT1 overexpression was also sufficient to partially rescue the inhibition of MCF7 xenograft growth caused by dietary S/G starvation, demonstrating that at least part of the effect of S/G starvation is tumor cell intrinsic (Figures 3M and 3N). These results indicate that PSAT1 expression is limiting for serine biosynthesis in luminal breast cancer cells and that low PSAT1 expression induces serine auxotrophy and sensitizes luminal breast cancer cells to S/G deprivation *in vitro* and *in vivo*.

**Figure 4.**
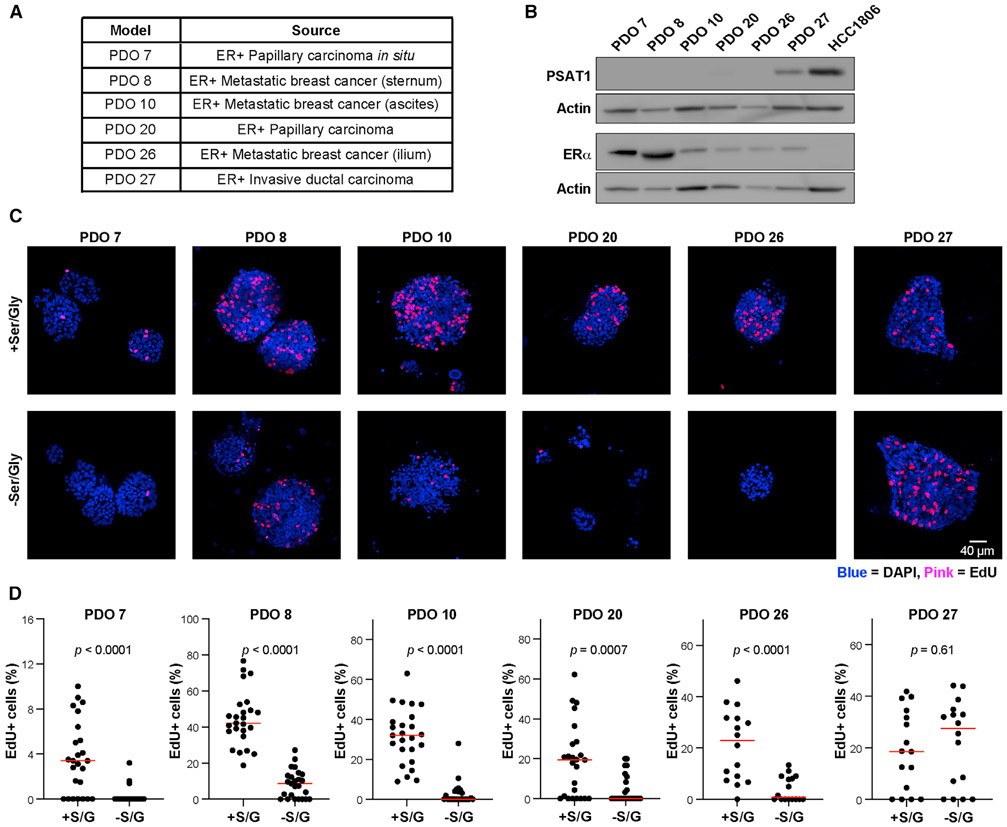
PSAT1 expression predicts sensitivity to serine starvation in patient-derived ER^+^ breast cancer organoids (A) Clinical characteristics of the 6 patient-derived organoid (PDO) models. (B) Western blot for ERα and PSAT1 in PDO models. HCC1806 basal breast cancer cell lysate is included as an ER^−^/PSAT1 high reference. (C) Representative confocal images of DAPI (blue) and EdU (pink) staining in PDO models treated with or without S/G for 10 days. (D) Quantification of EdU staining in PDO models. %EdU^+^ cells were calculated by dividing the number of EdU^+^ cells by the total number of cells in either 16 or 25 organoids per group. p values are from unpaired Student’s t tests.

These results suggest that PSAT1 expression likely predicts the sensitivity of luminal breast cancer cells to S/G starvation. To evaluate this possibility, we used six ER+ primary patient-derived organoid (PDO) models that we have established from core needle biopsies of both primary and metastatic tumors (Figure 4A). Consistent with luminal tumors and cell lines, five of the six PDOs expressed little or no PSAT1 (Figure 4B). Notably, 5-ethynyl-2′-deoxyuridine (EdU) incorporation revealed that S/G starvation causes a drastic reduction in proliferation in the five PSAT1-low PDOs, while proliferation of the lone PDO with high PSAT1 was not sensitive to S/G starvation (Figures 4C and 4D). These results support a model in which PSAT1 is the primary determinant of sensitivity to serine starvation in luminal/ER+ tumors.

### DNA methylation suppresses *PSAT1* in luminal breast tumors

**Figure 5.**
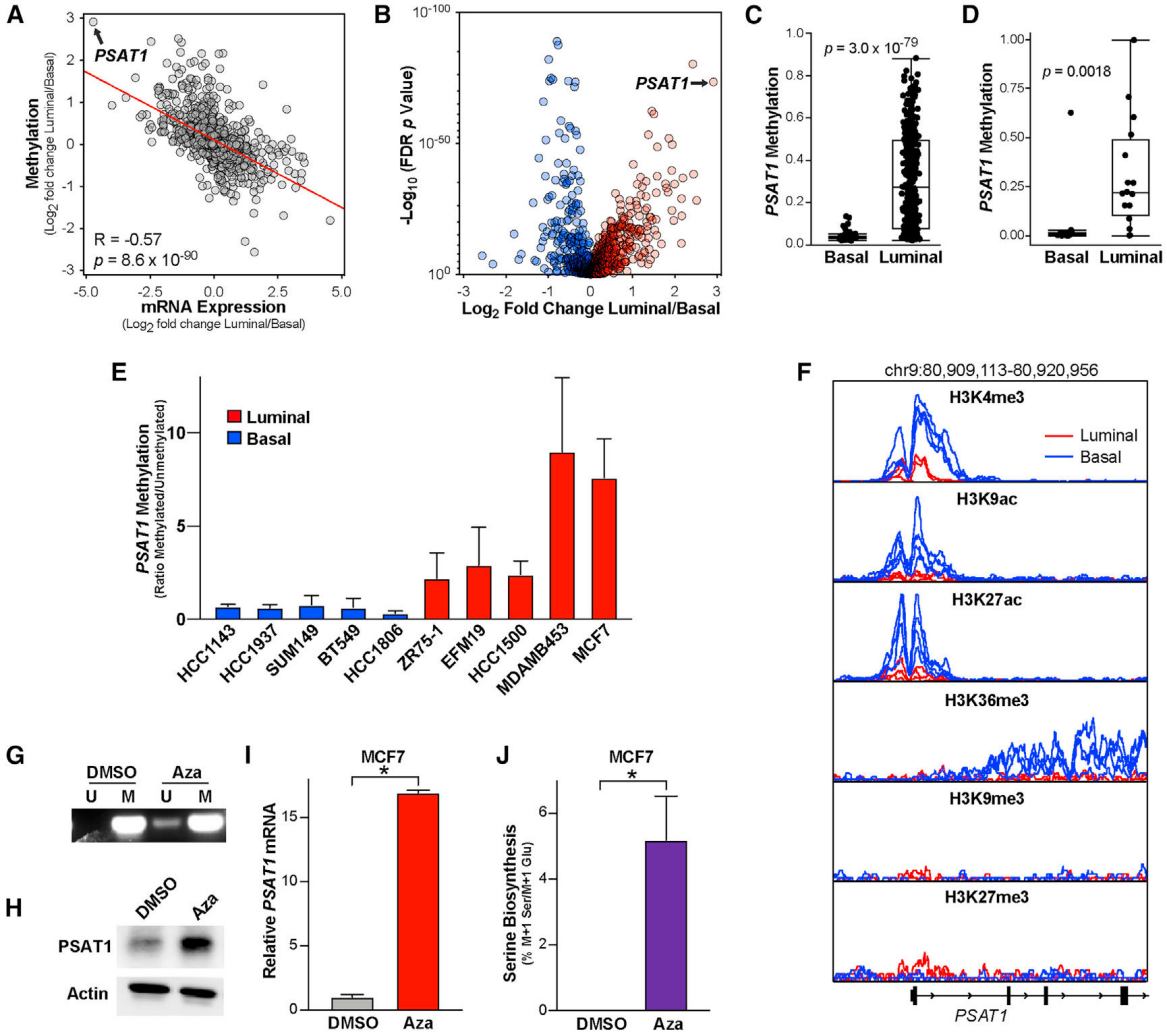
*PSAT1* is epigenetically silenced specifically in luminal breast cancer cells (A) Pearson correlation of fold changes in mRNA expression and DNA methylation between luminal and basal TCGA breast tumor samples. (B) Differences in metabolic gene methylation between luminal and basal breast tumors in the TCGA DNA methylation dataset. Data are the log_2_ fold change of mean gene methylation in luminal breast tumors relative to basal breast tumors. −Log_10_ p values from 2-sided Welch’s t tests that have been corrected for false discovery rate using the Benjamani-Hochberg method. (C) *PSAT1* DNA methylation in basal and luminal breast tumors in the TCGA dataset. p value from 2-sided Welch’s t test. (D) *PSAT1* DNA methylation in basal and luminal breast cancer cell lines in the CCLE DNA methylation dataset. p value from 2-sided Welch’s t test. (E) Quantitation of the ratio of methylated over unmethylated *PSAT1* DNA in basal and luminal breast cancer cells. Values are the means ± SEMs from 2–4 independent experiments. (F) Analysis of H3K4me3, H3K9ac, H3K27ac, H3K36me3, H3K9me3, and H3K27me3 ChIP-seq data from luminal (MCF7, ZR751,and MDAMB361, red) and basal (MDAMB231, MDMB436, MDAMB468, and HCC1937, blue) cell lines. Data are adapted from Franco et al. (2018). (G) Representative methylation-specific PCR detecting methylated (M) and unmethylated (U) *PSAT1* promoter DNA in MCF7 cells treated with azacytidine (5 μM) for 3 days. (H and I) PSAT1 protein (H) and mRNA (I) levels in MCF7 cells treated with azacytidine (5 μM for 3 days). Western blot is representative of 2 independent experiments. qPCR values are the means ± SDs of triplicate samples from an experiment representative of 3 independent experiments. *p < 0.05 in an unpaired 2-sided t test. (J) Serine biosynthesis in MCF7 cells treated with azacytidine (5 μM for 3 days). Values are the means ± SDs of triplicate samples from an experiment representative of 2 independent experiments. *p < 0.05 in an unpaired 2-sided t test.

Lineage-specific gene expression is often controlled by epigenetic modifications such as histone modifications and DNA methylation (Kim and Costello, 2017). As such, we analyzed the TCGA human breast tumor DNA methylation dataset, in which we saw a strong correlation between the differences in metabolic gene expression and methylation between luminal and basal tumors (Figure 5A). Importantly, *PSAT1* is the most differentially methylated metabolic gene between luminal and basal tumors (Figure 5B). DNA methylation is strongly correlated with the suppression of transcription, and high luminal *PSAT1* methylation is consistent with the low *PSAT1* seen in these tumors (Figures 1D and 5C) (Coloff et al., 2016). We also observed high *PSAT1* gene methylation specifically in luminal breast cancer cell lines using CCLE data and our own methylation-specific PCR assay (Figures 5D, 5E, and S4A). In addition to DNA methylation, we analyzed *PSAT1* chromatin immunoprecipitation sequencing (ChIP-seq) data for several histone H3 modifications in three luminal (MCF7, ZR751, and MDAMB361) and four basal (MDAMB231, MDMB436, MDAMB468, and HCC1937) breast cancer cell lines (Franco et al., 2018). As expected, these data revealed increased levels of active transcription, indicated by elevated H3K4me3, H3K9ac, H3K27ac, and H3K36me3 at or near the *PSAT1* transcriptional start site in basal cells relative to luminal (Figure 5F). However, the levels of the suppressive marks H3K9me3 and H3K27me3 were near background levels in both luminal and basal lines and were not strongly different between the two groups (Figure 5F). This suggested that DNA methylation may be the most relevant suppressive mechanism of *PSAT1* expression in luminal breast cancer cells. The treatment of MCF7 cells with the DNA methyltransferase (DNMT) inhibitor azacytidine was sufficient to strongly induce *PSAT1* mRNA and protein despite only partially reducing *PSAT1* gene methylation (Figures 5G-5I). Azacytidine was also sufficient to induce serine biosynthesis (Figure 5J). Treatment with another structurally distinct DNMT inhibitor (RG108) also induced *PSAT1* mRNA expression in MCF7 cells (Figure S4B), and azacytidine and the related DNMT inhibitor decitabine also induced *PSAT1* expression in MDAMB453 cells (Figure S4C). These results demonstrate that *PSAT1* gene methylation contributes to the reduced *PSAT1* expression and low serine synthesis observed in luminal cells.

### The low *PSAT1* phenotype of luminal tumors preexists in their cells of origin

**Figure 6.**
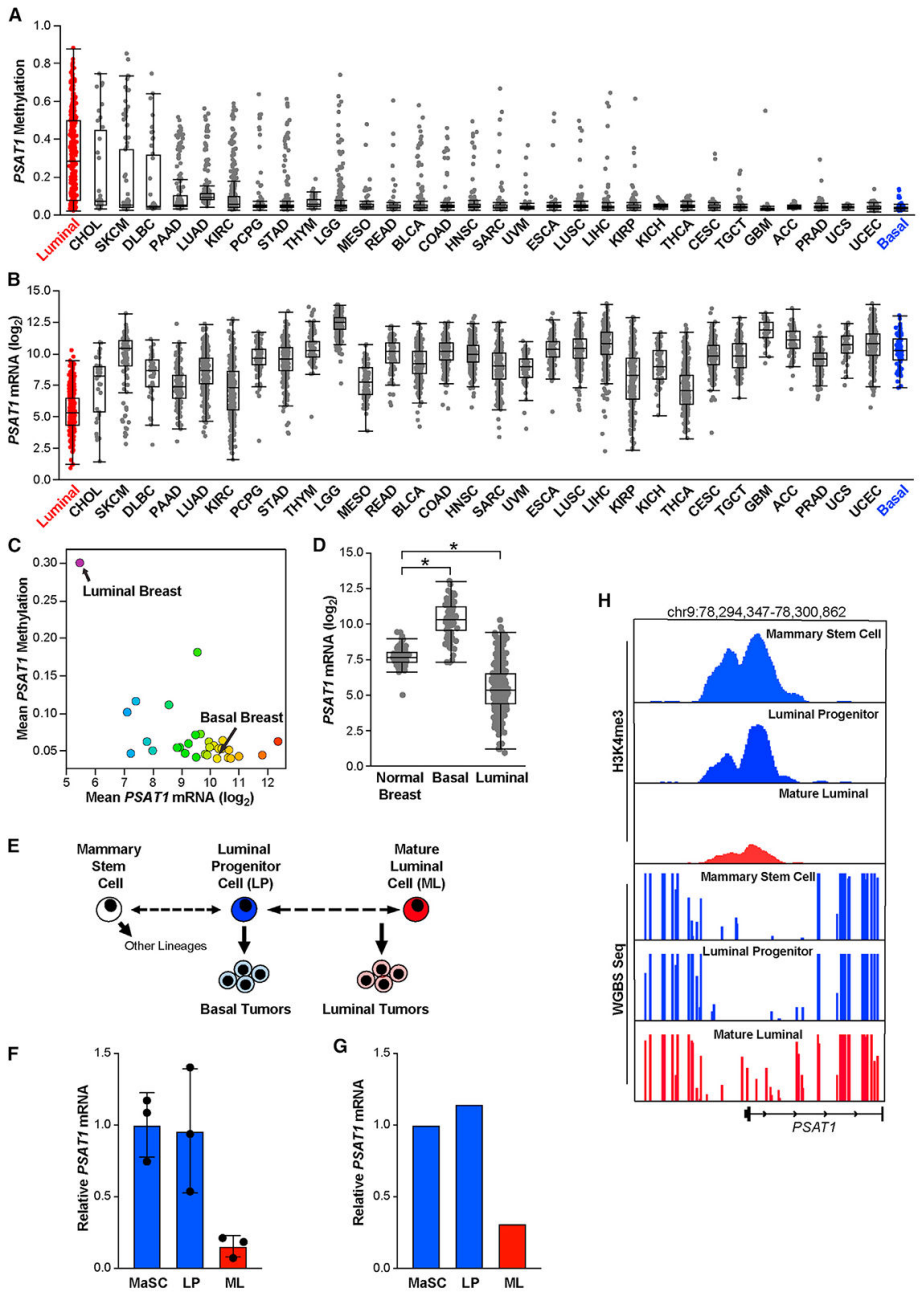
*PSAT1* tumor phenotypes preexist in luminal tumor cells of origin (A and B) *PSAT1* gene methylation (A) and mRNA expression (B) by tumor type in the TCGA Pan-Cancer Atlas datasets. TCGA study abbreviations listed in Figure 1A legend. (C) Mean *PSAT1* methylation and mRNA expression by tumor type in the TCGA Pan-Cancer Atlas datasets. (D) *PSAT1* mRNA levels in normal breast tissue relative to basal and luminal breast tumors in TCGA breast cancer RNA-seq data. *p < 0.05 in 2-sided Welch’s t tests. (E) Simplified model of the proposed mammary epithelial stem cell hierarchy. (F) Analysis of *PSAT1* mRNA expression in sorted mammary stem cells (MaSCs), luminal progenitor cells (LPs), and mature luminal cells (MLs) derived from normal human mammary glands. Microarray data adapted from Lim et al. (2009). *p < 0.05 in an unpaired 2-sided t test. (G) Analysis of *PSAT1* mRNA expression in sorted MaSC, LP, and ML cells. RNA-seq data adapted from Pellacani et al. (2016). (H) Analysis of *PSAT1* H3K4me3 ChIP-seq and whole-genome bisulfate sequencing data in sorted MaSC, LP, and ML cells. Data adapted from Pellacani et al. (2016).

To determine whether high *PSAT1* methylation may induce sensitivity to S/G deprivation in additional tumor types, we analyzed *PSAT1* methylation and gene expression in the TCGA Pan-Cancer Atlas dataset. Interestingly, luminal breast tumors have the highest *PSAT1* methylation and are the only tumor type to show consistently high *PSAT1* methylation, although outliers can be found in most tumor types (Figure 6A). Accordingly, luminal breast tumors have the lowest *PSAT1* mRNA expression of any tumor type (Figures 6B and 6C), suggesting that low *PSAT1*-induced sensitivity to S/G starvation may be a luminal breast tumor-specific vulnerability.

To understand how the low-*PSAT1* expression phenotype of luminal tumors arises, we investigated *PSAT1* expression in normal breast tissue. Interestingly, we found that normal breast expresses *PSAT1* at an intermediate level between that found in luminal and basal tumors (Figure 6D). This suggests that the low-*PSAT1* phenotype of luminal tumors represents either an active suppression of *PSAT1* or the outgrowth of a subpopulation of mammary epithelial cells that express low levels of *PSAT1*. To investigate the latter possibility, we examined the expression of *PSAT1* in two independent datasets of fluorescence-activated cell sorting (FACS)-sorted subpopulations of epithelial cells from normal breast reduction donors (Lim et al., 2009; Pellacani et al., 2016). The normal mammary gland is a complex structure that includes three major epithelial cell types–mammary stem cells (MaSC), luminal progenitor cells (LPs), and mature luminal cells (MLs) (Visvader and Stingl, 2014). It is believed that the cells of origin for basal tumors are similar to LPs and that the cells of origin for luminal tumors are more similar to MLs (Figure 6E). In two independent datasets, we found that *PSAT1* expression is significantly higher in MaSCs and LPs than in MLs (Figures 6F and 6G). We also observed increased H3K4me3 signal at the *PSAT1* transcription start site in MaSCs and LPs relative to MLs (Figure 6H). Importantly, whole-genome bisulfite sequencing data revealed increased DNA methylation near the *PSAT1* transcription start site in MLs than in LPs and MaSCs (Figure 6H). These data suggest that the *PSAT1* mRNA expression and DNA methylation phenotypes seen in basal and luminal tumors preexist in their respective cells of origin in the normal mammary gland and is consistent with a model in which the serine auxotrophy of luminal tumors is caused by an outgrowth of luminal cells with an already-low *PSAT1* phenotype.

### Serine auxotrophy can also be induced by *PHGDH* methylation

**Figure 7.**
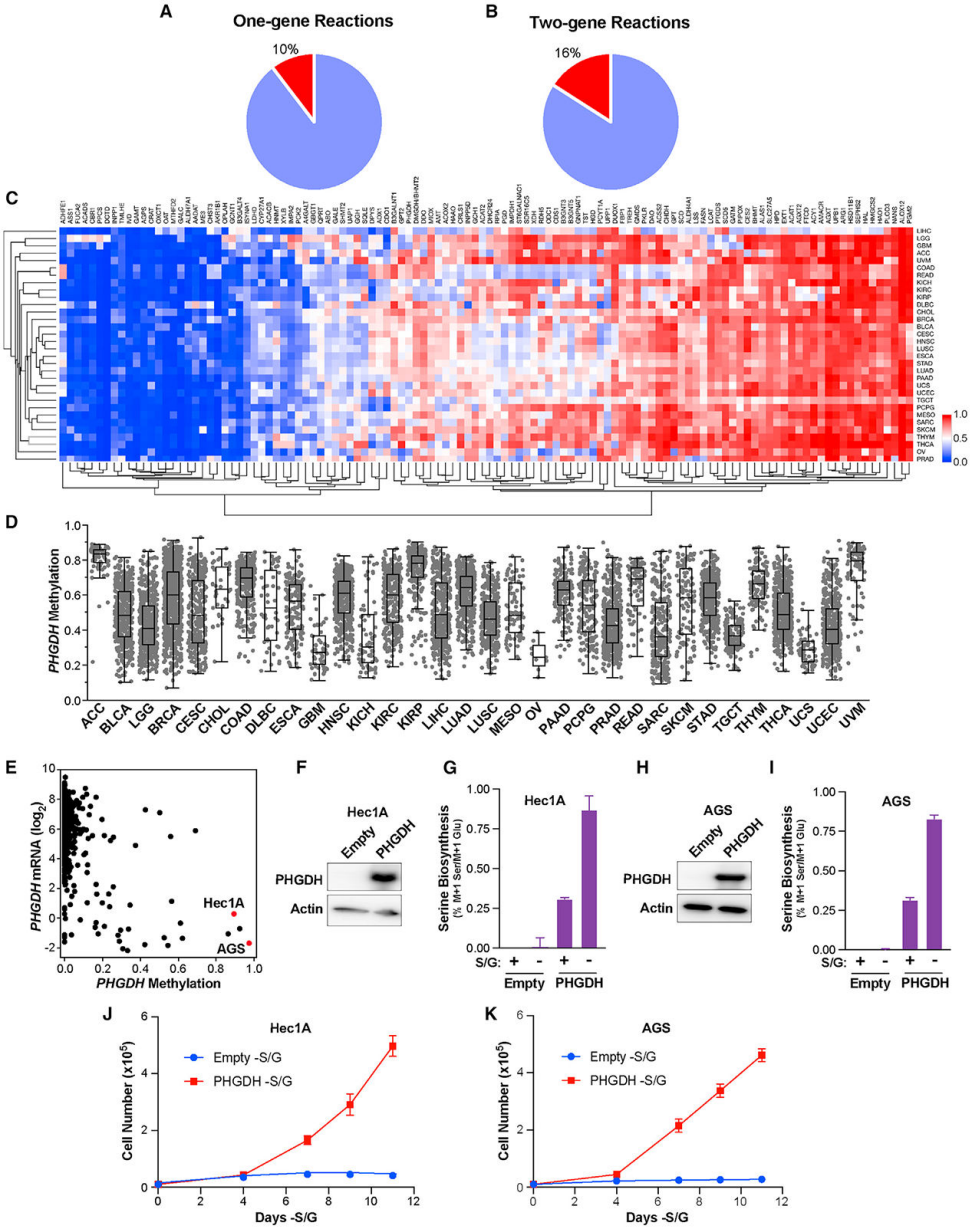
*PHGDH* hypermethylation can also induce serine auxotrophy (A and B) Fraction of 1-gene (A) and 2-gene (B) reactions from the KEGG database that contain a highly methylated metabolic gene. (C) Hierarchical clustering of mean metabolic gene methylation values from the TCGA Pan-Cancer Atlas dataset. TCGA study abbreviations listed in Figure 1A legend. (D) Methylation of *PHGDH* in the TCGA Pan-Cancer Atlas dataset. (E) PHGDH DNA methylation and mRNA expression data from the CCLE. (F–I) Western blot for PHGDH and serine biosynthesis in empty vector and PHGDH-overexpressing Hec1A (F and G) and AGS (H and I) cells. Values are the means ± SDs of triplicate samples from an experiment representative of 2 independent experiments. (J and K) Growth curves of empty vector and PHGDH-overexpressing Hec1A (J) and AGS (K) cells growing in the absence of S/G. Values are the means ± SDs of triplicate samples from an experiment representative of 2 independent experiments.

We have identified a vulnerability in serine metabolism induced by *PSAT1* gene methylation specifically in luminal tumors. This suggests that studying lineage-specific metabolic gene methylation may be an effective method of identifying vulnerabilities in specific subtypes of tumors. To identify additional potential vulnerabilities, we analyzed the Pan-Cancer Atlas DNA methylation dataset for metabolic genes that met three criteria: (1) DNA methylation correlated strongly with gene expression, (2) methylation is variable across tumor types, and (3) the median gene methylation was at least as high as *PSAT1* methylation in luminal tumors. In total, 289 of the 1,454 metabolic genes we analyzed fit this profile. To focus on methylation events that could induce a vulnerability, we analyzed the Kyoto Encyclopedia of Genes and Genomes (KEGG) database for metabolic reactions that are mediated by enzymes encoded by only one or two metabolic genes, reasoning that the hypermethylation of one-gene reactions could lead to the limitation of a particular metabolic pathway (similar to *PSAT1*) and that hypermethylation of one gene in a two-gene reaction could lead to a collateral lethality vulnerability, similar to those induced by metabolic gene deletion (Muller et al., 2015). Of the 803 one-gene reactions and 211 two-gene reactions in the KEGG database, 10% and 16%, respectively, were associated with a methylation event (Figures 7A and 7B). The patterns of these methylation events were varied, with some showing high methylation in most tumor types, some with low methylation in most tumors, and others that were highly variable across tumor types (Figure 7C). Interestingly, among these was *PHGDH*, the methylation of which was highly variable across tumor types (Figure 7D). To determine whether *PHGDH* hypermethylation may also induce serine auxotrophy, we acquired two cell lines–Hec1A (endometrial cancer) and AGS (stomach cancer)–both of which display high *PHGDH* methylation and low PHGDH mRNA expression in CCLE data (Figure 7E). These cell lines both express undetectable PHGDH protein (Figures S5A and S5B) and do not proliferate in the absence of S/G (Figures S5C and S5D). Importantly, the overexpression of PHGDH in Hec1A and AGS cells was sufficient to induce serine biosynthesis and proliferation without S/G (Figures 7F-7K). These results suggest that in addition to *PSAT1*, the hypermethylation of *PHGDH* can induce serine auxotrophy and that any tumor exhibiting high methylation of *PHGDH* or *PSAT1* (and possibly *PSPH*) may be auxotrophic for serine and sensitive to therapies targeting serine availability.

## DISCUSSION

Initial efforts of targeting serine metabolism in breast cancer were focused on “gain-of-function” cases in which the amplification of *PHGDH* leads to elevated rates of serine biosynthesis in basal tumors. However, it is becoming clear that targeting gain-of-function metabolic alterations in cancer can be challenging if redundant pathways remain present and active (Méndez-Lucas et al., 2020). This seems to be particularly true for serine metabolism, in which in most circumstances serine derived from the biosynthetic pathway and taken up from circulation are each sufficient to promote tumor growth (Montrose et al., 2021; Méndez-Lucas et al., 2020; Tajan et al., 2021). Because our goal was to identify “loss-of-function” cases, in which pathway redundancy is limited by lineage-specific gene expression, we have identified a potentially more tractable opportunity to target serine metabolism in luminal, not basal, breast cancer, in which serine biosynthesis is naturally limited by the hypermethylation of the *PSAT1* gene.

While targeting serine availability is of significant interest in a variety of tumor types, it is important to note that there could be negative side effects of such therapies. It was recently observed that long-term dietary serine starvation can lead to sensory defects in mice (Gantner et al., 2019), and therefore, it remains unclear whether altering dietary serine content alone can form the basis of an effective treatment in humans. Other approaches, such as the inhibition of serine transporters or enzymatic degradation of plasma serine may also be effective treatments for exploiting serine auxotrophy. Interestingly, hypermethylation of the *PSAT1* gene in luminal breast tumors is reminiscent of T cell acute lymphoblastic leukemia (T-ALL), in which hypermethylation of the asparagine synthetase gene sensitizes T-ALL cells to degradation of plasma asparagine with l-asparaginase (Worton et al., 1991).

It is also important to note that the local availability of nutrients in tumors is variable across tumor types and can be affected by a variety of factors, including diet, stromal cells, and the extent of vascularization (Muir et al., 2017; Sullivan et al., 2019a). Importantly, it has recently been shown that serine availability may be uniquely low in the mammary gland relative to other tissues (Sullivan et al., 2019b). A low serine environment combined with our discovery of serine auxotrophy in luminal breast tumors may provide a therapeutic window in which serine metabolism can be effectively targeted without negative side effects in luminal/ER+ breast cancer

While PHGDH has traditionally been viewed as the rate-limiting step of serine biosynthesis, our data demonstrate that PSAT1 is limiting for serine biosynthesis in luminal breast cancer cells. These findings suggest that in addition to PHGDH, PSAT1 is an important and regulated step in serine biosynthesis in cancer cells. PSAT1 regulation has been shown to be important in other tumor types, including in pancreatic cancer, in which modulation of PSAT1 expression in LKB1-mutant tumors influences epigenetics and gene expression (Kottakis et al., 2016). Serine metabolism has also been suggested to play a role in the response of ER+ breast cancer cells to endocrine therapy (Metcalf et al., 2021), suggesting a potential role for PSAT1 in drug response. These and other studies highlight the continued importance of serine metabolism in tumors, both as a contributor to cancer biology and a potential therapeutic vulnerability.

### Limitations of the study

For this study, we made use of several luminal and basal breast cancer cell lines, which we found to robustly maintain the differential PSAT1 expression phenotype that we observed in human breast tumors. Importantly, however, these cell lines do not show differential expression of PHGDH, which is also observed in human tumors. This leaves open the possibility that low PHGDH could also contribute to a serine auxotrophy phenotype in luminal breast cancer patients, and our data demonstrate that low PHGDH can induce serine auxotrophy in other types of cancer cells. While this limitation of our breast cancer cell line models may simplify our mechanistic understanding of luminal serine auxotrophy, low PSAT1 and low PHGDH would likely result in an even stronger serine auxotrophy phenotype in human breast tumors.

## STAR★METHODS

### RESOURCE AVAILABILITY

#### Lead contact

Further information and requests for resources and reagents should be directed to and will be fulfilled by the lead contact, Jonathan Coloff (coloff@uic.edu).

#### Materials availability

Materials generated in this study will be made available by the lead contact upon request.

#### Data and code availability

- Data: This paper analyzes existing, publicly available data. The accession numbers and sources of this data are listed in the Quantification and Statistical Analysis section.
- Code: This paper does not report original code.
- Any additional information required to reanalyze the data reported in this paper is available from the lead contact upon request.

### EXPERIMENTAL MODEL AND SUBJECT DETAILS

#### Mouse procedures

All mouse procedures were approved by the University of Illinois at Chicago Animal Care Committee. HCC1806 (1 × 10^6^), MCF7 (3 × 10^6^), MCF7-EMPTY (5 × 10^6^) and MCF7-PSAT1 (5 × 10^6^) cells were injected into the fat pad of the #4 mammary glands of 6 to 8-week-old athymic nude-foxn1_nu_ female mice (Envigo). Cell suspensions were injected in a volume of 50 μL growth factor-reduced Matrigel (Corning). Estrogen (E2) was administered via silastic capsules implanted subcutaneously as previously described (Molloy et al., 2014). Even though not required for HCC1806 tumor growth, E2 was provided for all experiments to maintain constant conditions. All tumors were monitored by caliper measurements over time and tumor volume was calculated using the formula ½ (width^2^ x length). Mice were euthanized according to institutional guidelines. Serine and glycine free (TD.160752) and control (TD.110839) diets were purchased from Envigo and diet formulations are listed as reported (Sullivan et al., 2019b). Custom diets were administered five days after surgery and replaced at least weekly.

#### Standard cell culture and media

Hec1A, AGS, and HCC1500 cells were acquired from ATCC. All other cell lines (HCC1806, SUM149, BT549, HCC1937, HCC70, BT20, MCF7, MDAMB453, EFM19, ZR75-1, and T47D) were acquired from the Brugge Lab at Harvard Medical School, and were authenticated by STR analysis. All cell lines were of female origin. Cell lines were regularly tested for mycoplasma using the MycoAlert Mycoplasma Detection Kit (Lonza). Cells were grown in human plasma-like medium according to the published formulation (Cantor et al., 2017) with 5% dialyzed FBS (Sigma) and Pen/Strep (Invitrogen) at 37 degrees C with 5% CO_2_. Media was changed at least every two days. As needed, cells were incubated in RPMI media (R9010-01, US Biological Life Sciences) with or without serine and glycine with 5% dialyzed FBS and Pen/Strep. Cells were counted using a Z1 Coulter Particle Counter (Beckman Coulter) and growth rates were calculated using the following formula: growth rate = ln(final cell number/initial cell number)/time.

#### Patient-derived breast cancer organoid cultures

Patient-derived breast cancer organoids (PDOs) were cultured as previously described (Sachs et al., 2018). Briefly, PDOs were resuspended in Cultrex growth factor-reduced BME type 2 (Trevigen, 3533-001-02), and plated in 50 μL drops on a 24 well plate. 500 μL of PDO media (Sachs et al., 2018) was added to each well and changed every 3-4 days. PDO medium was supplemented with 5 μM Y-27632 for the first 3 days of organoid formation. PDO cultures were passaged approximately every 2 weeks. For passaging, PDOs were incubated in 1× Dispase solution with 2 mg/mL collagenase for 45 minutes at 37°C, and mechanically disrupted by passing through a 26G needle. PDO fragments were seeded in Cultrex at 1:2 to 1:4 ratio.

### METHODS DETAILS

#### Western blots

Cells were lysed in mammalian cell lysis buffer (50mM Tris pH 7.5, 150mM NaCl and 0.5% NP40) containing a protease and phosphatase inhibitor cocktail (Bimake.com) and 1 μM MG132 (Selleckchem). To generate PDO protein lysates, organoids were incubated for 1 h on ice in Cell Recovery solution (Corning, 354253) and lysed using RIPA buffer (Boston Bioproducts) supplemented with protease and phosphatase inhibitors (Roche). Protein concentration was determined by BCA assay (Thermo Fisher). Quantified protein samples were separated by electrophoresis on 4–20% ready-made Tris-Glycine gels (Invitrogen) and transferred to PVDF membranes (Millipore). Membranes were blocked with 2% bovine serum albumin for 1 h and incubated overnight with one or more primary antibodies: PSAT1 (Thermo Fisher, PA5-22124.), PHGDH (Sigma, HPA021241), PSPH (Santa Cruz, sc-365183), ERα (Cell Signaling, 8644) and Actin (Sigma, A1978). Overexpression and knockout of PSAT1 (Figure 3) confirmed the correct band on PSAT1 western blots. Membranes were washed with tween 20-containing tris buffered saline and incubated with fluorescence- or HRP-conjugated secondary antibodies (Bio-Rad). Rhodamine-conjugated anti-tubulin was also treated as a secondary antibody (Bio-Rad, 12004166). Images were detected using a ChemiDoc MP Imaging System (Bio-Rad).

#### RT-qPCR

RNA was isolated using Trizol reagent (Thermo Fisher) and cDNA was generated using qScript cDNA Synthesis Kits (Quantabio). RT-qPCR was performed with SYBR Green on an ABI ViiA7 real-time PCR system (Applied Biosystems), and results were normalized to the expression of *RPLPO*. The primer sequences were*: PSAT1-F: 5′-CGGTCCTGGAATACAAGGTG-3′; PSAT1-R: 5′-AACCAAGCCCATGACGTAGA -3′; PHGDH-F:5′-ATCTCTCACGGGGGTTGTG-3′; PHGDH-R: 5′-AGGCTCGCATCAGTGTCC-3′; PSPH-F: 5′-TGGAGATGGTGCCACAGATA-3′; PSPH-R: 5′-CCTCCAAATCCAATGAAAGC-3′ and RPLPO-F: 5′-ACGGGTACAAACGAGTCCTG-3′ and RPLPO-R: 5′-CGACTCTTCCTTGGCTTCAA-3′*.

#### GC-MS metabolite analyses

Cells were incubated in media containing α-^15^N-glutamine (Cambridge Isotope Laboratories) for 24 hours. Cells were lysed on ice in methanol, water, and chloroform. Norvaline was used as an internal standard. For mouse plasma analysis, plasma was mixed with isotopically labeled amino acid standards (Cambridge Isotopes) for absolute quantification. Plasma metabolites were extracted by adding HPLC grade ethanol (Sigma-Aldrich), vortexing, and centrifuging at 21000 × g at 4°C for 10 min. All extracts were air dried and derivatized with MOX (Thermo Fisher, PI45950) and *N*-*tert*-butyldimethylsilyl-*N*-methyltrifluoroacetamide with 1% *tert*-butyldimethylchlorosilane (Sigma-Aldrich). Samples were analyzed by GC/MS using a HP-5MS Ultra Inert GC column (19091S-433UI, Agilent Technologies) installed in an Agilent 7890B gas chromatograph coupled to an Agilent 5977B mass spectrometer. Helium was used as the carrier gas. One microliter of sample was injected at 280°C. After injection, the GC oven was held at 60°C for 1 min. The oven was then ramped to 320°Cat 10°C/min and held for 9 min at 320°C. The MS system operated under electron impact ionization mode at 70 eV and the MS source and quadrupole were held at 230°C and 150°C respectively. Mass isotopomer distributions were determined by manually integrating ion fragments using ChemStation software (Agilent). Natural abundance correction was performed in R or in MATLAB. Total abundance was normalized to the norvaline internal standard and to cell number counted in proxy wells. Serine biosynthesis was calculated by determining the fraction of labeled (M+1) serine and dividing it by the fraction of M+1 glutamate.

#### Knockout and overexpression

Knockout of PHGDH and PSAT1 were performed using lentiCRISPR v2 Puro (Addgene, 52961). The following oligos were cloned into BsmBI cut lentiCRISPR v2 sgLuc F: 5′- caccgGAGGCTAAGCGTCGCAA-3′; sgLuc R: 5′- aaacTTGCGACGCTTAGCCTCc-3′; sgPSAT1-1 F: 5′- caccgACCGAGGGGCACTCTCGG-3’; sgPSAT1-1 R: 5′- aaacCCGAGAGTGCCCCTCGGTc-3′; sgPSAT1-2 F: 5′- caccgCATCACGGACAATCACCA-3′; sgPSAT1-2 R: 5′- aaacTGGTGATTGTCCGTGATGc-3′; sgPHGDH F: 5 ′-caccgAGTCTGGCCAGTGTGCCG-3′; sgPHGDH R: 5′-aaacCGGCACACTGGCCAGACTc-3′. For PSAT1 and PHGDH overexpression, human PSAT1 or PHGDH were cloned into pLenti CMV Neo DEST (Addgene, 17392) using the Gateway cloning system (Thermo Fisher). The PSAT1 K200A mutant was generated using the QuickChange II XL Site-Directed Mutagenesis kit (Agilent, 200521). To generate lentiviral particles, HEK293T cells were transduced with PAX2, VSVG, and the lentiviral plasmid of interest using polyethylenimine (Polysciences). Viral supernatants were collected 48 and 72 hours after transduction. After infection with polybrene (hexadimethrine bromide, Sigma), cells were drug selected until mock infected cells were completely cleared after which antibiotic was removed.

#### Methylation-specific PCR

Bisulfite conversion of genomic DNA was carried out using the EZ DNA Methylation-Gold kit (ZYMO Research Corporation) according to the manufacturer’s instructions. The following MSP primers were designed using MethPrimer 2.0 (Li and Dahiya, 2002): methylated *PSAT1* F 5′-GTAGGGTTTGCGATAGTACGG-3′; methylated *PSAT1* R 5′-GCTACGATAAAAATCTACAACCGAC-3′; unmethylated *PSAT1* F 5′-GGGTTTGTGATAGTATGGGT-3′; unmethylated *PSAT1* R 5′-CCACTACAATAAAAATCTACAACCAAC-3′. MSP PCR conditions consisted of a denaturing step of 15 min at 95°C followed by 40-50 cycles of 30s at 95°C, 30s at 59°C and 30s at 72°C, with a final extension of 7 min at 72°C. PCR products were analyzed by running on a 2% agarose gel with SYBR-Safe DNA gel stain (Invitrogen).

#### Breast cancer tissue processing for PDO generation

Core needle biopsies from primary or metastatic breast cancer were obtained during standard diagnostic procedure at BIDMC/Dana-Farber-Harvard Cancer Center. Informed consent was obtained as per Federal Regulations (45 CFR 46), BIDMC IRB Guidelines, and requirements of HIPAA. The protocol was approved by Dana-Farber/Harvard Cancer Center Institutional Review Board (IRB #17-627). Biopsies were cut into small fragments and digested in 10mL of PluriSTEM Dispase-II solution (Sigma, SCM133) supplemented with 2 mg/mL Collagenase (Sigma, C9407) on an orbital shaker at 37°C for 45-60 minutes. The digested tissue was sequentially sheared using 10 mL and 5 mL plastic pipettes, and the collected fractions were strained over a 100 μm filter. 2% FBS was added to the suspension, and the breast fragments were centrifuged for 5 minutes at 400 rcf. The pellet was resuspended in 10 mL of AdDF+++ (Advanced DMEM/F12 supplemented with 10 mM HEPES, 2mM Glutamine and antibiotics), and centrifuged for 5 minutes at 400 rcf. Breast cancer cells obtained from pleural effusion were washed 5-6 times AdDF+++ to remove erythrocytes.

#### SG starvation and EdU labeling of PDO cultures

PDOs were seeded on 4-well cell culture chamber slides (Corning, 354114) at equal numbers. PDO media was replaced with vehicle or serine and glycine-free media the next day and incubated for 10 days. Serine and glycine-free Advanced DMEM/F12 was generated using amino acid, glucose, and pyruvate free DMEM/F12 (US Biological, D9807-11) supplemented with ethanolamine, glutathione, ascorbic acid, transferrin, and AlbuMAX II to the levels found in commercial Advanced DMEM/F12. PDO cultures were treated with 10 μM 5-ethynyl-2′-deoxyuridine (EdU) for 4h and fixed for 40 minutes with 4% PFA. The fixed PDOs were permeabilized with 0.5% Tx-100 for 20 minutes and labeled using the Click-iT EdU Alexa Fluor 488 imaging kit (ThermoFisher, C10337) according to manufacturer’s instructions. Labeled PDOs were imaged with a Zeiss LSM880 confocal microscope. The ratio of EdU+ cells was quantified in 16 to 25 organoids/group by counting the number of EdU+ cells per total number of cells. Statistical analysis was performed with Prism GraphPad. Student’s t-test was used to calculate p values.

### QUANTIFICATION AND STATISTICAL ANALYSIS

#### Bioinformatics and public datasets

TCGA Pan-Cancer Atlas RNAseq (EB++AdjustPANCAN_IlluminaHiSeq_RNASeqV2.geneExp.xena), DNA methylation (jhu-usc.edu_PANCAN_HumanMethylation450.betaValue_whitelisted.tsv.synapse_download_5096262.xena), and copy number (broad.mit.edu_PANCAN_Genome_Wide_SNP_6_whitelisted.gene.xena) data files were downloaded from the University of California, Santa Cruz Xena browser (xena.ucsc.edu). TCGA study abbreviations are: LUAD = lung adenocarcinoma, LUSC = lung squamous cell carcinoma, PRAD = prostate adenocarcinoma, UCEC = uterine corpus endometrial carcinoma, BLCA = bladder urothelial carcinoma, TGCT = testicular germ cell tumors, ESCA = esophageal carcinoma, PAAD = pancreatic adenocarcinoma, KIRP = kidney renal papillary cell carcinoma, LIHC = liver hepatocellular carcinoma, CESC = cervical squamous cell carcinoma and endocervical adenocarcinoma, SARC = sarcoma, THYM = thymoma, MESO = mesothelioma, COAD = colon adenocarcinoma, STAD = stomach adenocarcinoma, KIRC = kidney renal clear cell carcinoma, THCA = thyroid carcinoma, HNSC = head and neck squamous cell carcinoma, BRCA = breast invasive carcinoma, READ = rectum adenocarcinoma, SKCM = skin cutaneous melanoma, LGG = brain lower grade glioma, PCPG = pheochromocytoma and paraganglioma. Low detection genes, defined as >5% tumors with 0 counts, were removed from the analysis. CCLE RNAseq and DNA methylation data was downloaded from the CCLE web portal (portals.broadinstitute.org/ccle) and the DepMap portal (depmap.org/portal). METABRIC data was downloaded from cBioPortal (cbioportal.org). PAM50 subtype calls for breast tumors and cell lines were obtained from UCSC Xena (TCGA.BRCA.sampleMap/BRCA_clinicalMatrix) (Jiang et al., 2016). Cancer cell line ChIP-seq data was downloaded from NCBI-GEO (accession number GSE85158) and converted to the BigWig format with the *rtracklayer* package (v1.42.2) in R3.5.2. Sorted mammary epithelial cell ChIP-seq and whole genome bisulfite sequencing data was downloaded from the CEERHRC Network (http://www.epigenomes.ca/).

#### Statistical analyses

For basal vs luminal analyses, luminal A and B samples were grouped together and compared to basal samples using two-sided Welch’s t-tests with correction for false discovery rate using the Benjamani-Hochberg method, while HER2+ and normal-like samples were excluded. For correlation of methylation and expression of metabolic genes (Gaude and Frezza, 2016), the DNA methylation probe that most strongly anti-correlated with RNAseq levels for that gene (lowest Pearson’s R) was selected and compared using Pearson’s correlation. Genomics data was visualized using the Integrated Genomics Viewer version (v2.4.10). Hierarchical clustering and heatmap generation were performed using R 3.5.1 or Morpheus (https://software.broadinstitute.org/morpheus). A shell script provided by B. Arman Aksoy and Chris Sander (Aksoy et al., 2014) was used to parse KEGG to identify reactions involving one or two genes. Data for heatmaps are log_2_ median centered. Boxplots, volcano plots, and scatterplots were generated using JMP Pro 12 and GraphPad Prism 8. Statistical analyses were performed using JMP Pro 12, GraphPad Prism 8, and Microsoft Excel. Where applicable, the Benjamini-Hochberg procedure was used to correct for false discovery rate. Error bars represent either the SD or SEM as described in the figure legends. The Geiser-Greenhouse correction was used for two-way repeated measures ANOVA and linear mixed model analyses.

## Supplementary Material

1

## Figures and Tables

**Table:** KEY RESOURCES TABLE

REAGENT or RESOURCE	SOURCE	IDENTIFIER
Antibodies		
PSAT1	Thermo Fisher	Cat# PA5-22124; RRID:AB_11153526
PHGDH	Sigma	Cat# HPA021241; RRID:AB_1855299
PSPH	Santa Cruz	Cat# sc-365183; RRID:AB_10709319
ERα	Cell Signaling	Cat# 8644; RRID:AB_2617128
Actin	Sigma	Cat# A1978; RRID:AB_476692
Tubulin	Bio-Rad	Cat# 12004165; RRID:AB_2884950
Chemicals, peptides, and recombinant proteins		
MOX	Thermo Fisher	PI45950
TBDMS (*N*-*tert*-butyldimethylsilyl-*N*-methyltrifluoroacetamide with 1% *tert*-butyldimethylchlorosilane)	Sigma	375934
Experimental models: Cell lines		
Hec1a	ATCC	HTB-112
AGS	ATCC	CRL-1739
HCC1500	ATCC	CRL-2329
HCC1806	Brugge Lab	N/A
SUM149	Brugge Lab	N/A
BT549	Brugge Lab	N/A
HCC1937	Brugge Lab	N/A
HCC70	Brugge Lab	N/A
BT20	Brugge Lab	N/A
MCF7	Brugge Lab	N/A
MDAMB453	Brugge Lab	N/A
EFM19	Brugge Lab	N/A
ZR75-1	Brugge Lab	N/A
T47D	Brugge Lab	N/A
Experimental models: Organisms/strains		
Hsd: athymic nude-foxn1^nu^ female mice	Envigo	N/A
Control diet	Envigo	TD.110839
Serine and glycine free diet	Envigo	TD.160752
Oligonucleotides		
Primers for PSAT1, PHGDH, PSPH, and RPLPO (see RT-qPCR section of STAR Methods)	This paper	N/A
Oligos used to generate sgRNAs targeting PSAT1 and PHGDH (see Knockout and Overexpression section of STAR Methods)	Sequences from Park et al., Nat Genetics, 2016.	PMID: 27992415
Methylation specific PCR primers (see Methylation Specific PCR section of STAR Methods)	This paper	N/A
Recombinant DNA		
lentiCRISPR v2 Puro	Addgene	52961
pLenti CMV Neo	Addgene	17392

## Highlights

- Luminal breast tumors are auxotrophic for serine
- Luminal serine auxotrophy is caused by lineage-specific hypermethylation of *PSAT1*
- *PSAT1* suppression preexists in the luminal tumor cells of origin
- Serine auxotrophy sensitizes to dietary serine starvation
