# Supplementary material for: Lineage-specific silencing of PSAT1 induces serine auxotrophy and sensitivity to dietary serine starvation in luminal breast tumors
Source: Cell Rep. Author manuscript; Available in PMC 2022 Feb 15. (PMC8845302; doi:10.1016/j.celrep.2021.110278)
Supplement: 1 [file NIHMS1772781-supplement-1.pdf]

Cell Reports, Volume *38*

Supplemental information

Lineage-specific silencing of *PSAT1* induces serine auxotrophy and sensitivity to dietary serine starvation in luminal breast tumors

Bo-Hyun Choi, Vipin Rawat, Jenny Högström, Philippa A. Burns, Kelly O. Conger, Mete Emir Ozgurses, Jaymin M. Patel, Tejas S. Mehta, Angelica Warren, Laura M. Selfors, Taru Muranen, and Jonathan L. Coloff

Supplemental Figure 1

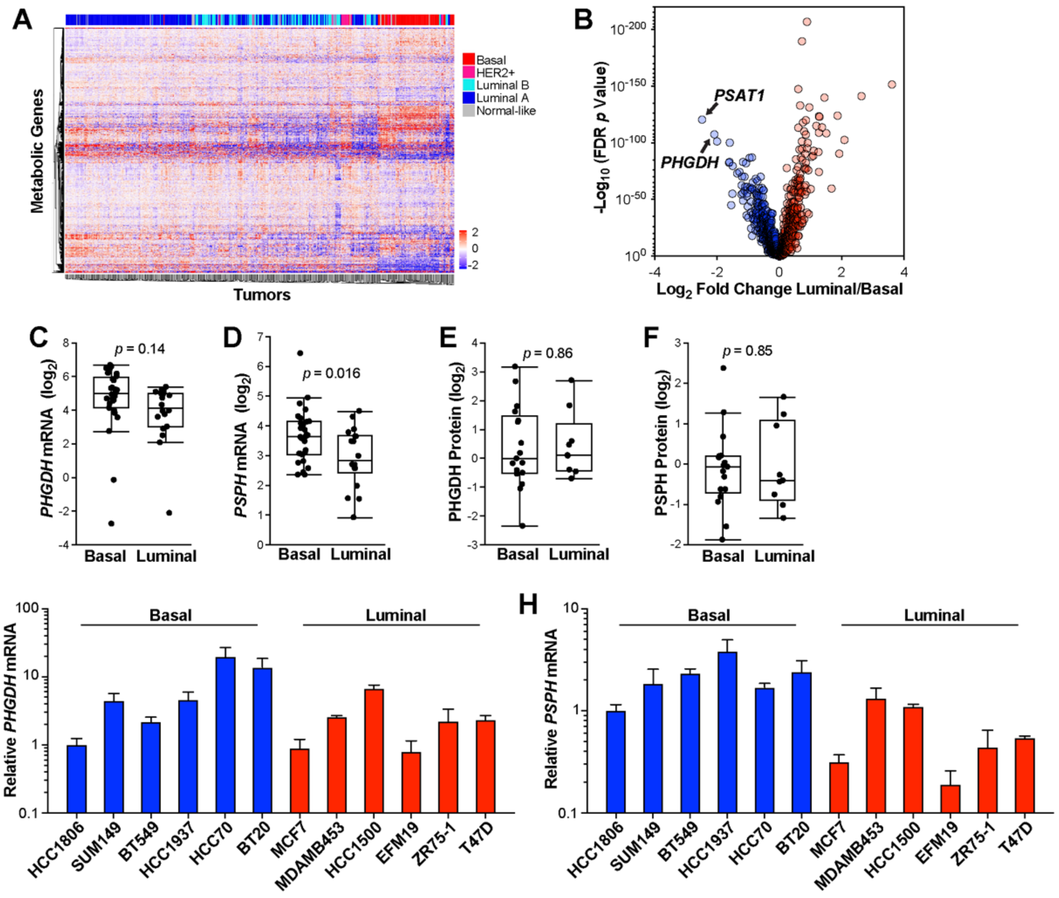

Supplemental Figure 1. Serine Synthesis Pathway Gene Expression in Basal and Luminal Breast Cancer. Related to Figure 1.

(A) Hierarchical clustering of human breast tumors from the TCGA Pan-Cancer Atlas data set by metabolic gene expression. Data are log2-median centered.

(B) Differences in metabolic gene expression between luminal and basal breast tumors in the METABRIC data set. Data is the log2 fold change of mean gene expression in luminal breast tumors relative to basal breast tumors. -Log10 p values from two-sided Welch's t tests that have been corrected for false discovery using the Benjamani-Hochberg method.

(C & D) *PHGDH* **(C)** and *PSPH* **(D)** mRNA levels in basal and luminal breast cancer cell lines from the Cancer Cell Line Encyclopedia (CCLE). *p* values from two-sided Welch's t tests.

(E & F) PHGDH **(E)** and PSPH **(F)** protein levels in basal and luminal breast cancer cell lines from the Cancer Cell Line Encyclopedia (CCLE). *p* values from two-sided Welch's t tests.

(G & H) *PHGDH* **(G)** and *PSPH* **(H)** mRNA levels in basal and luminal breast cancer cell lines. Values are the means ± SEM of three independent experiments.

Supplemental Figure 2

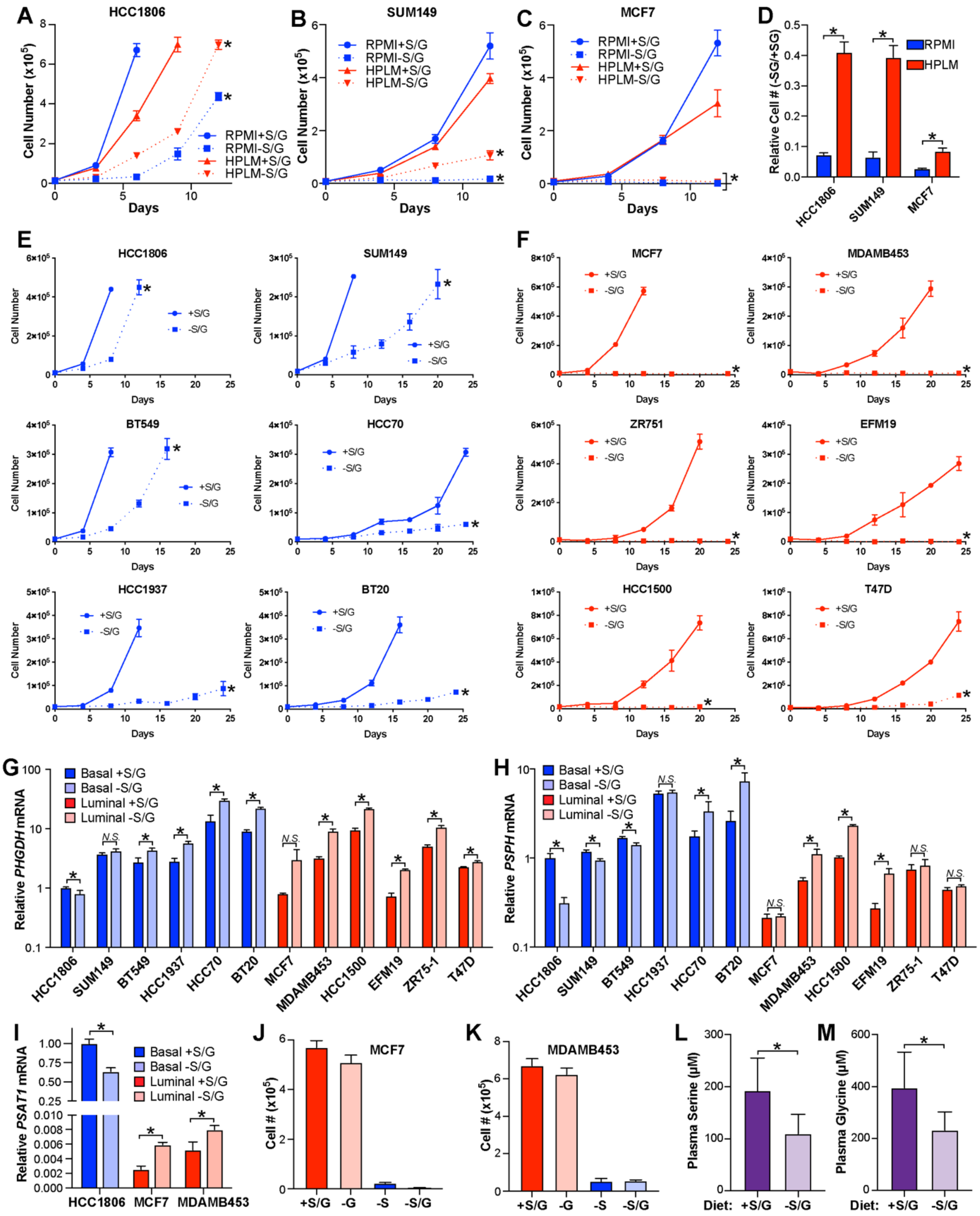

Supplemental Figure 2. Basal and Luminal Breast Cancer Cell Sensitivity to Serine and Glycine Starvation. Related to Figure 2.

(A – C) Growth curves of HCC1806 **(A)**, SUM149 **(B)**, and MCF7 **(C)** cells cultured in RPMI or HPLM in the presence or absence of S/G. * indicates $p < 0.05$ in two-way repeated measures ANOVA tests comparing +S/G to –S/G samples.
(D) Ratio of cell numbers after 6 (HCC1806) or 8 (SUM149, MCF7) days of culture -S/G relative to +S/G in RPMI (blue) or HPLM (red). * indicates $p < 0.05$ in an unpaired two-sided t test.
(E & F) Growth curves of basal **(E)** and luminal **(F)** breast cancer cells cultured in HPLM media ±S/G. Values are the means ± SD of one experiment representative of three independent experiments. * indicates $p < 0.05$ in two-way repeated measures ANOVA tests.
(G & H) *PHGDH* **(G)** and *PSPH* **(H)** mRNA levels in basal and luminal lines treated +/- S/G for 48 hrs. Values are the mean ± SD of triplicate samples from an experiment representative of three independent experiments. * indicates $p < 0.05$ from unpaired two-sided t tests. *N.S.* (not significant) indicates $p > 0.05$.
(I) *PSAT1* mRNA levels in breast cancer cells cultured +/- S/G for 30 days. Values are the mean +/- SD of triplicate samples from an experiment representative of two independent experiments.
(J & K) MCF7 **(J)** or MDAMB453 **(K)** cell number after 10 days of culture +/- serine, glycine, or both. Values are the mean ± SD of triplicate samples.
(L & M) Serine **(L)** and glycine **(M)** concentrations in mouse plasma after 21 days on custom +/- S/G diets. n = 5 mice. * indicates $p < 0.05$ in an unpaired two-sided t test.

Supplemental Figure 3

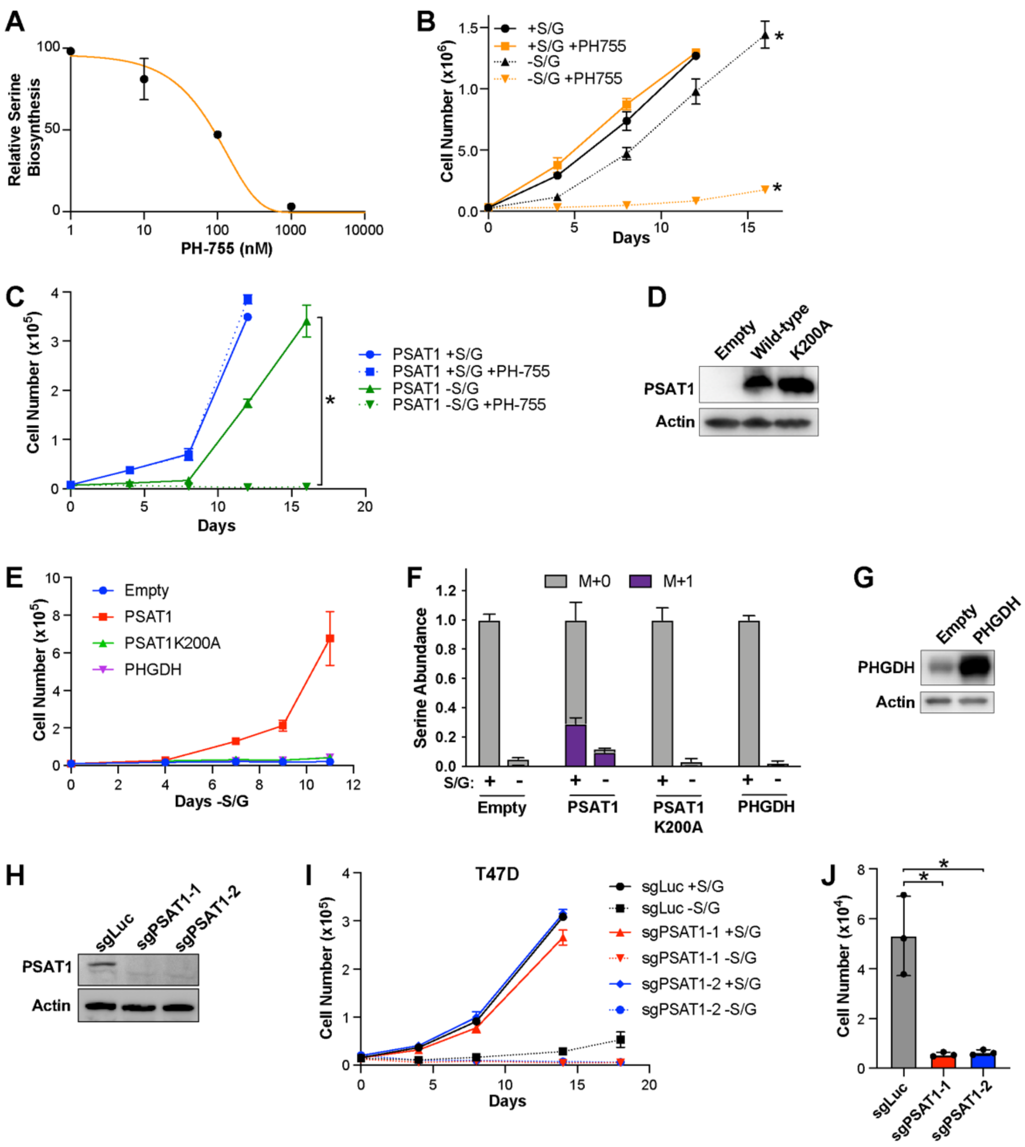

Supplemental Figure 3. Serine Synthesis Pathway Redundancy in Breast Cancer Cells. Related to Figure 3.

(A) Relative labeled serine levels after culture with PH-755 at the indicated doses. Values are the mean ± SD of triplicate samples from an experiment representative of two independent experiments.

(B) Proliferation of HCC1806 cells treated +/- 1 µM PH-755 +/- S/G. Values are the mean ± SD of triplicate samples from an experiment representative of three independent experiments. * indicates p <0.05 in two-way repeated measures ANOVA. -S/G data is compared to corresponding +S/G data.

(C) Growth curve of PSAT1 overexpressing MCF7 cells treated +/- 1 μM PH-755 and S/G. Values are the mean ± SD of triplicate samples.
(D) Western blot for PSAT1 in empty vector, wild-type PSAT1, or PSAT1 K200A overexpressing MCF7 cells.
(E) Growth curve in the absence of S/G of MCF7 cells overexpressing either empty vector, wild-type PSAT1, PSAT1 K200A mutant, or PHGDH. Values are the mean ± SD of triplicate samples from an experiment representative of two independent experiments.
(F) Serine abundance and biosynthesis in empty vector, wild-type PSAT1, PSAT1 K200A, or PHGDH overexpressing MCF7 cells treated +/- S/G for 48 hrs. M+1 (in purple) indicates "heavy" serine made in the serine synthesis pathway. Values are the mean ± SD of triplicate samples from an experiment representative of two independent experiments.
(G) Western blot for PHGDH in empty vector and PHGDH overexpressing MCF7 cells.
(H) Western blot for PSAT1 in control (sgLuc) and PSAT1 knockout (sgPSAT1-1 and sgPSAT1-2) T47D cells.
(I) Growth curve of control (sgLuc) or PSAT1 knockout (sgPSAT1-1 and sgPSAT1-2) T47D cells treated +/- S/G. Values are the mean ± SD of triplicate samples from an experiment representative of two independent experiments.
(J) Cell number from the final time point of -S/G samples from **(I)**. * indicates $p < 0.05$ in unpaired Student's t tests.

Supplemental Figure 4

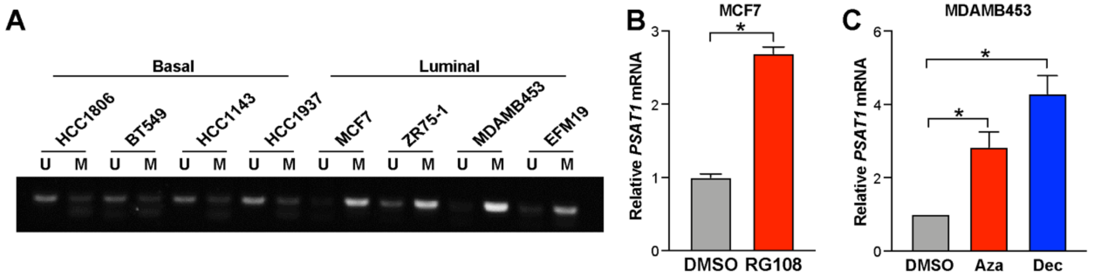

Supplemental Figure 4. *PSAT1* Methylation in Breast Cancer Cells. Related to Figure 5.

(A) Representative methylation specific PCR detecting methylated (M) and unmethylated (U) *PSAT1* promoter DNA in basal and luminal breast cancer cells**.**

(B) *PSAT1* mRNA in MCF7 cells treated with RG108 (10 μM for 3 days). Values are the mean +/- SD of triplicate samples from an experiment representative of three independent experiments. * indicates $p < 0.05$ in an unpaired two-sided t test.

(C) PSAT1 mRNA level in MDAMB453 cells treated with azacytidine (5 μM for 3 days) or decitabine (1 μM for 3 days). Values are the mean ± SD of triplicate samples from an experiment representative of two independent experiments. * indicates $p < 0.05$ in an unpaired two-sided t test.

Supplementary Figure 5

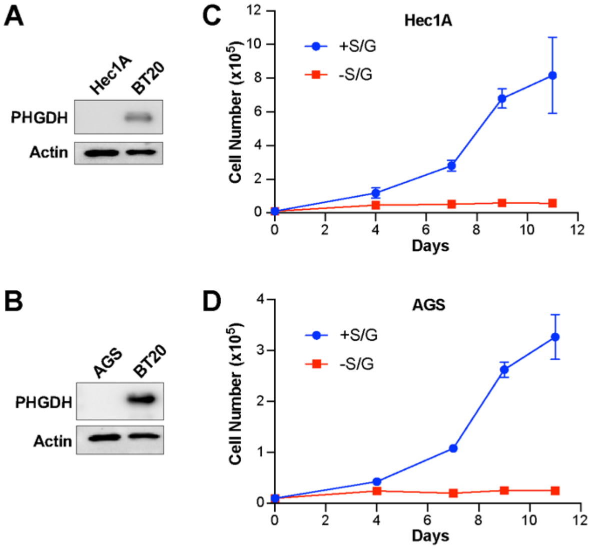

Supplemental Figure 5. Cancer Cell Lines with Low PHGDH Expression are Sensitive to Serine and Glycine Starvation. Related to Figure 7.

(A – D) Western blots for PHGDH **(A, B)** and growth curves +/- S/G **(C, D)** in Hec1A and AGS cells. BT20 basal breast cancer cell lysate is included as a PHGDH-high reference. Growth curve data are the mean ± SD of triplicate samples from an experiment representative of two independent experiments.
